# Pattern Recognition Receptors (PRRs) in Macrophages Possess Prognosis and Immunotherapy Potential for Melanoma

**DOI:** 10.3389/fimmu.2021.765615

**Published:** 2021-11-09

**Authors:** Qihang Zhao, Qiang Wang, Tengjiao Wang, Junfang Xu, Tingting Li, Qiuyan Liu, Qinghua Yao, Pin Wang

**Affiliations:** ^1^ National Key Laboratory of Medical Immunology, Institute of Immunology, Navy Medical University, Shanghai, China; ^2^ Beijing Friendship Hospital, Capital Medical University, Beijing, China; ^3^ Centre for Translational Medicine, Navy Medical University, Shanghai, China; ^4^ Shanghai Pulmonary Hospital, Tongji University School of Medicine, Shanghai, China; ^5^ College of Medical Technology, Shanghai University of Medicine & Health Sciences, Shanghai, China; ^6^ Department of Integrated Chinese and Western Medicine, Cancer Hospital of University of Chinese Academy of Science, Institute of Cancer Research and Basic Medical Sciences of Chinese Academy of Sciences, Zhejiang Cancer Hospital, Hangzhou, China; ^7^ Key Laboratory of Traditional Chinese Medicine Oncology, Zhejiang Cancer Hospital, Hangzhou, China

**Keywords:** pattern recognition receptors, skin cutaneous melanoma, prognosis, immune-infiltration, macrophage

## Abstract

**Background:**

Pattern recognition receptors (PRRs) family plays a vital role in the initial stage of innate immune response and the subsequent activation of adaptive immunity. Increasing evidences have indicated that several PRRs play critical roles in the progress of inflammation and tumorigenesis. However, the comprehensive significance of PRRs family in clinical prognosis of different cancers is still elusive.

**Methods:**

We analyzed expression of 20 canonical PRRs in tumor samples from 9502 patients of 33 tumor types. Next, we used expression profiles of PRRs in skin cutaneous melanoma (SKCM) to build a Cox prognosis model. Then, we analyzed immune infiltration features and immune activity of high risk score and low risk score patients. Finally, we analyzed the single-cell sequencing data of different cancers and detected the expression of PRRs in mouse melanoma model to identify PRRs-expressing cell types.

**Results:**

We found PRRs had a significantly positive correlation with prognosis in SKCM rather than other tumors, and PRR-based Cox model had a much better prognosis potential than any single PRR. Further analysis shows risk score could indicate immunocyte infiltration and immune activity in SKCM. We also found the expressions of some PRR genes were highly correlated with the expression of immune checkpoints molecules in SKCM, indicating they could be indicators for clinical immune therapy. Finally, we found only in SKCM samples, the expression of PRRs is especially high in a subpopulation of macrophages with a trait of CD206 low expression, probably explaining why PRRs have prognosis potential in melanoma.

**Conclusions:**

Our study reveals PRR family in macrophages has a positive prognosis potential in melanoma and could be valuable for clinical prognosis and immune therapy.

## Introduction

Pattern recognition receptors (PRRs) family, including Toll-like receptors (TLRs), Nod-like receptors (NLRs), and other types of nucleic acid sensors, play a vital role in the initial stage of innate immune response (1). After recognizing pathogen-associated molecular patterns (PAMPs) or danger-associated molecular patterns (DAMPs), PRRs are activated and then trigger downstream immune signaling pathways, such as IRF3/7 and NF-κB signals to mediate the activation of innate immune system and subsequent adaptive immune responses, leading to the clearance of invading pathogens or transformed cells in human body. Thus, PRRs are of high relevance to multiple physiological and pathological processes, and the aberrant expression or dysfunction of PRRs are implicated in generation and progress of diverse types of diseases, such as infection, autoimmunity diseases and cancers (2).

Substantial evidences have indicated that inflammation plays critical driving roles on tumor progress and metastasis, and several regulation mechanisms of PRR family genes in tumor proliferation and progression have been demonstrated in the past few years (3). On one hand, PRRs can promote the formation of cancer, for example, alveolar epithelial TLR3 can be activated by tumor exosomal RNAs to promote formation of lung pro-metastatic niche (4); nuclear cyclic GMP-AMP synthase (cGAS, MB21D1) can suppress DNA repair and promote tumorigenesis (5); up-regulation of TLR2 induced by signal transducer and activator of transcription 3 (STAT3) can promote gastric tumorigenesis that independent of tumor inflammation (6). On the other hand, several PRRs also have been proved to inhibit tumor progression, such as deficiency of retinoic acid-inducible gene-I (RIG-I) can promote hepatocellular carcinoma (HCC) carcinogenesis (7). Yet, the key question remains whether the expression of PRR family genes have clinical prognosis correlations in certain types of tumors and even could be prognosis markers are still not fully understood.

In this study, we analyzed the correlations between PRR genes and prognosis of many different cancers and found PRR genes highly positive corelated with the prognosis of SKCM. Therefore, we proposed a prognosis model with risk score based on PRRs expression, and analyzed the feature of immunocyte infiltration within different risk groups and their correlation with expression of immune checkpoint genes. Through analysis of single-cell sequencing dataset, we investigated the expression of PRR genes in different cellular types on SKCM samples and found PRRs were mainly expressed in macrophages with a feature of CD206-low expression. This work substantially expands the understanding of PRRs in tumor prognosis, leading to recognition of their clinical application potentials.

## Material and Methods

### Pan-Cancer Expression Analysis and Prognosis Analysis of Multiple Genes by GEPIA2

Gene Expression Profiling Interactive Analysis 2 (GEPIA2) is a website-based analysis platform, which collects and integrates the transcriptome expression profiles and survival data of diverse tumor types from the Cancer Genome Atlas (TCGA) database and other tumor database. For expression analysis, first, select “Multiple Gene Comparison” interface, next, input interested gene lists and select interested types of cancer, and then get the result of expression profiles. For prognosis analysis, first, select “Survival map” interface, next input interested gene lists and interested types of cancer, and select method as overall survival, significance level as 0.05, P-value adjustment as no adjustment, group cutoff as medium, then get the result of survival maps.

### Gain of Transcriptome Data and Clinical Data of Patients

Melanoma transcriptome profiling (RNAseq) data that harmonized to fragments per kilobase million (FPKM) were downloaded from TCGA (https://tcga-data.nci.nih.gov/tcga/) of the SKCM project and GEO dataset (GSE65904) (https://www.ncbi.nlm.nih.gov/geo/). Clinical data of patients with melanoma were also received from the SKCM project of TCGA and GEO dataset (GSE65904).

### Establishment of the PRR-Based Cox Model to Evaluate the Risk Score

The PRR-based Cox model was established by R package “survival”. The Cox proportional hazards model was the model to investigate the relationship of predictors and the time-to-event (8). It assumes that the predictors have a multiplicative effect on the hazard and that this effect is constant over time, i.e.,


h(t|x)=h0(t)exp(β1x1+⋯+βpxp),

where *h(t|x)* is the hazard at time t for a subject with a set of predictors *x1, …, xp*, *h0(t)* is the baseline hazard function, and *β1, …, βp* are the model parameters describing the effect of the predictors on the overall hazard. Based on this principle, first, single variable Cox proportional risk regression analysis was performed to confirm 15 PRRs significantly correlated to overall survival (OS) in TCGA SKCM dataset(P<0.05). Next, multivariable Cox proportional risk regression analysis was carried out to establish the prognosis model of SKCM (PRR-based risk model), 8 of PRRs were included in this Cox proportional hazards model, and Cox regression coefficient (*β*) of 8 PRRs was calculated. We used the following formula to calculate the risk score of each patient:


$$risk\;score(S_{k},X_{i})=exp(X_{i}\beta ),$$

in which, *Xi* is the expression of gene *i* in the sample *k*, the variable *β* denotes the vector of Cox regression coefficient of the survival analysis corresponding to the *Xi*, and risk score of each samples is the sum of 8 PRR’s risk score. The median risk score was determined as the critical value to divide the SKCM dataset into high-risk and low-risk. To determine the role of risk score in predicting the clinical prognosis of GC patients, Kaplan–Meier Plotter was drawn to clarify the difference of survival time between high-risk group and low-risk group.

### Validation of the PRR-Based Cox Model

To confirm the credibility of PRR-based risk model, we calculated the receiver operating characteristic (ROC) curve and calculated the areas under curve (AUC). Next, to further investigate whether risk score can be used as an independent prognosis predictor in TCGA dataset of SKCM patients, single variable and multivariate Cox regression analysis were conducted. Age, gender, stage, grade, T, N, M, and risk score were used as covariates. Combined with clinical data, chisquare tests are performed to calculate the correlations between the risk score, expression of PRRs with clinicopathological characteristics.

### Analysis of the Correlation Between Risk Score and the TNM Staging

Firstly, the clinical data and results of risk score of 468 SKCM patients were coordinated. Next, the risk score or the expression of 8 PRRs were summarized in several groups with different TNM stages. Non-significance results (P>0.05) were filtered. Results were displayed on box-plots graph utilizing ggplot2 R packages.

### Analysis of Tumor Purity in SKCM Samples by ESTIMATE Algorithm

The calculation of tumor purity, immune score, stromal score, and ESTIMATE score were analyzed by ESTIMATE algorithm with ESTIMATE R package (9). The results were visualized utilizing ggplot2 R packages, and their R values were calculated by R package of ggplot2.

### Correlation Analysis Between Immunocyte Infiltration and PRR-Based Risk Score

Immune-cell infiltration of SKCM samples from TCGA were downloaded from TIMER database (10), Correlation analysis between immunocyte infiltration and risk score were analyzed under the environment of R. First, upload the file of risk score of SKCM patients which were calculated by Cox model, and then perform the correlation test between risk score and immune-cell infiltration, including neutrophil, macrophage, DC, CD8^+^T cell, and CD4^+^T cell. The results were visualized by R package of ggplot2.

### Tumor Immune Infiltration Composition and Immune Activity Analysis by TIP

Tumor immune infiltration composition and immune activity analysis of 20 lowest risk score SKCM samples and 20 highest risk score samples were analyzed by tracking tumor immunophenotype (TIP) algorithm (http://biocc.hrbmu.edu.cn/TIP/) (11). Firstly, according to the rank of risk score, the transcriptome data of 20 lowest risk score SKCM samples and 20 highest risk score samples were selected and then uploaded to the TIP website, respectively. After analysis, the calculation results and statistical graphs were obtained from the website.

### Gene Expression Correlation Analysis of SKCM by GEPIA2

Utilizing GEPIA2, briefly, “correlations” interface was selected, and next, selected genes were inputted and expression datasets such as “SKCM Tumor” were selected. “Correlation coefficient” was set up as “Pearson” and the result of expression correlation were obtained.

### Collection of Single-Cell Transcriptome Data

Single-cell transcriptome data from different types of tumors including SKCM (GSE72056) (12), BRCA (GSE176078), and PAAD (GSE111672) (13) were downloaded from GEO database, and LIHC single-cell data were downloaded from CNGB database (CSE0000008) (14) (https://db.cngb.org/search/project/CNP0000650/). The details of these data were shown in **Table S4**.

### Single-Cell Data Processing

We used reads count matrix as input, the single cell data preprocessing and unsupervised clustering was performed based on the Seurat R package (v.4.0.3) (15). The scale factor was set as 10000 and natural-log transform was used for normalization. We excluded the genes detected in less than 10 cells for downstream analysis and only kept the protein coding genes. The principal component analysis (PCA) was performed for detecting the highly variable genes. The top 20 PCs were selected with a resolution parameter equal to 0.8. Nonlinear dimensionality reduction techniques t-distributed stochastic neighbor embedding (t-SNE) and uniform manifold approximation and projection (UMAP) was used to depict the distribution of cell groups’ cluster. We continued to use the definition for cell type provided by original research of the cancer data cohort.

### Risk Score Definition in Single-Cell Analysis

We defined the celluar PRR risk score formula to test the risk level of each cell, for the k-th cell *C_k_*, the risk score is:


$$\text{Risk score}(C_{k},X_{i})=\text{exp}(X_{i}\beta )$$

in which, *X_i_* is the expression of gene i in the cell k, the variable *β* denotes the vector of Cox regression coefficient of the survival analysis coresponding to the *X_i_*.

### Single-Cell Differential Gene Detection

We used the mean value of risk score as the cutoff to divide the SKCM-infiltrated macrophage cells into two groups (PRR high and PRR low groups). For the two different groups, we used the Seurat Findall Markers function to test genes with fold difference more than 0.25 and detectable expression in more than 25% of cells in either of the two populations. The cell distribution and gene expression comparisons between two groups were performed using WilCox rank-sum tests and Student’s t test respectively. All statistical analyses and presentation were performed using R (v.4.0.3).

### Enrichment Analysis of Melanoma-Infiltrated Macrophages

R package functional annotation clusterprofiler was used to perform GO and KEGG enrichment analysis on the up-regulated and down-regulated genes of high risk and low risk melanoma-infiltrated macrophages groups (16). In this process, the critical value of the significant gene functions and pathways to be screened was set as P value < 0.05. Next, we imported gene expression data into GSEGO function in the R package for enrichment analysis, the number of permutations was set as 1000, and other parameters were set as the default settings. To analyze the results, we selected the pathway of gene enrichment with a normal p-value < 0.05 and FDR q-value < 0.25.

### Mice, Cell Lines and Isolation of Tumor-Infiltrated Immunocytes

Melanoma cell line B16 cells were injected into subcutaneous of C57/B6-L mice (n=3), after injection of 18 days, tumors were dissected from the surrounding fascia, mechanically minced, and treated with DNase I (50µg/ml, Sigma) and collagenase P (2 mg/ml, Sigma) for 10 min at 37°C. Tumor-infiltrating immunocytes were enriched using an OptiPrepTM density gradient (Sigma, Catalog #07820), followed by CD45^+^, CD19^+^, or CD3^+^ MACS positive selection (Miltenyi). CD45^+^ cells were sorted on a FACS Aria II (BD Biosciences) to obtain CD11b^+^ F4/80^+^ cells, or NK1.1^+^ cells.

### Extraction of Total RNA and Quantitative Real Time PCR (RT-PCR) Assay of PRRs

Total RNA of isolated cells was extracted with TRIzol reagent (Thermo Fisher Scientific) according to manufacturer’s instruction, and 0.1μg total RNA was reverse transcribed with High Efficient Reverse Transcription Kit (Toyobo). SYBR RT-PCR kit (Takara) and LightCycler (Roche) were used for quantitative RT-PCR analysis as described (17). Data were normalized to GAPDH expression, and the negative control was set to a value of 1.

### Statistics

The statistical significance of expression of genes in low risk and high risk score group was analyzed with unpaired t test in GraphPad Prism version 8.0.1. Other statistical analyses were performed with R (v.4.0.3), and a p-value < 0.05 was considered significant.

## Results

### Expression of PRR Family Is Highly Positive Correlated to Prognosis in SKCM

**Figure 1:**
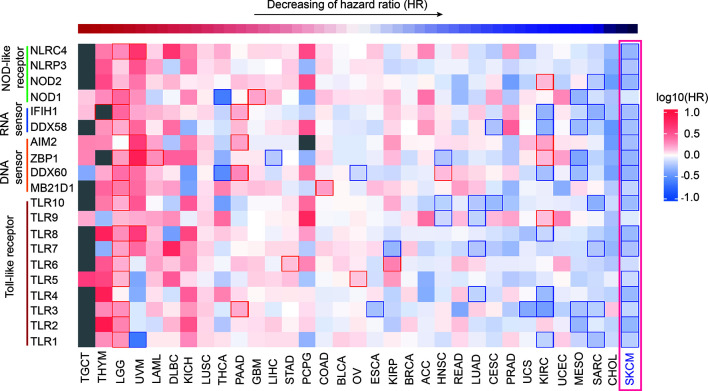
Pan-cancer prognosis analysis of PRRs. Pan-cancer prognosis analysis of PRRs by GEPIA2 shows PRRs family have the specific positive prognosis ability in SKCM (33 tumor types, 9502 samples, the square frame of some samples means P value <0.05 in this prognosis analysis of the indicated PRR).

Utilizing GEPIA2 tumor database (18), we comprehensively analyzed the transcriptome data of 9502 patients of 33 tumor types from The Cancer Genome Atlas (TCGA) database. We selected 20 canonical PRR genes as targets to analyze their correlations with tumor prognosis, including TLR family, NLR family, DNA sensors and RNA sensors (19, 20). Interestingly, results shows the expression of PRR family is specific high positive-correlated to prognosis in SKCM (**Figure 1** and **Table S1**), while the expression itself has no significant difference in SKCM compared with other tumors (**Figure S1**). Survival curve of each PRR is shown in **Figure S2**, and 17 out of 20 PRR genes significantly positive correlated to prognosis in SKCM (log10HR <0, pHR <0.05), indicating the expressions of PRR family genes have important implications for SKCM prognosis.

On the other hand, we also found the expression of PRR family was specifically highly negatively correlated to prognosis in brain lower grade glioma (LGG), and 15 PRRs are significant negative correlated to prognosis (log10HR>0, P<0.05), indicating the expressions of PRRs may also play critical regulation roles for LGG patients’ survival. It is also worth noting that expression of PRRs significantly correlates to survival times in several types of cancers, such as NOD2, IFIH1, and TLR1/3/7/10 significantly positively correlate to prognosis in sarcoma (SARC); NOD1, DDX58, ZBP1, DDX60, and TLR2/3 significantly positively correlate to prognosis in mesothelioma (MESO); DDX58, AIM2, DDX60, and TLR3 significantly positively correlate to prognosis in pancreatic adenocarcinoma (PAAD). As pan-cancer survival analysis shows the expression of PRR genes most correlates to prognosis of SKCM. Next, we focused on the prognosis potentials of PRRs in SKCM.

### Establishment of PRR-Based Prognosis Model in SKCM

**Figure 2:**
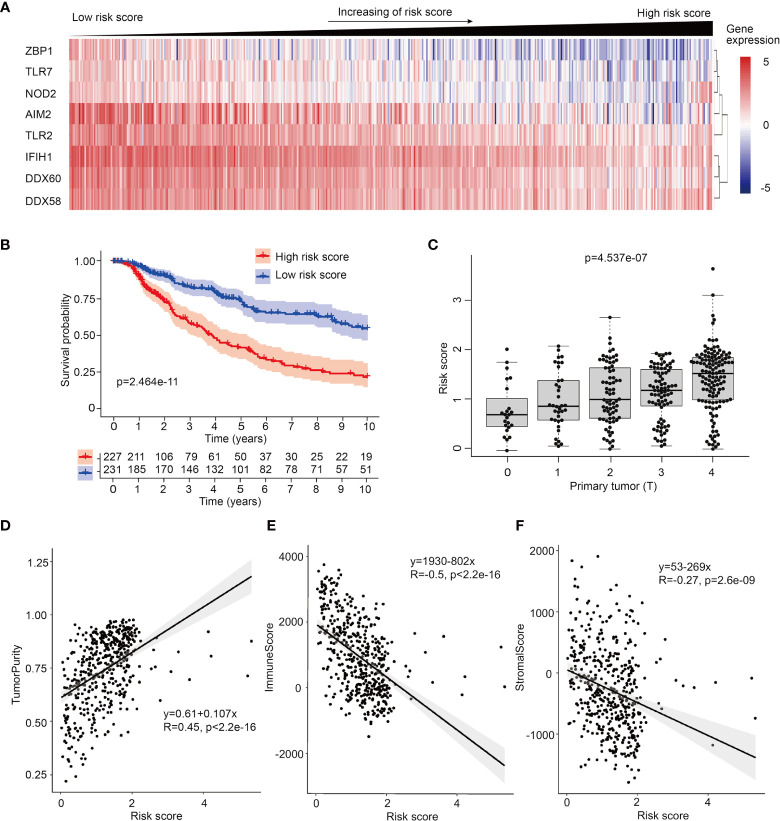
Establish of PRR-based prognosis model in SKCM. **(A)** Heatmap of 8 PRR molecules’ expression used in the prognosis model in 468 SKCM patients ranked by their risk score. **(B)** Survival curve of low risk score group and high risk score group of SKCM patients, samples which no data of survival times are filtered out. **(C)** Risk score is high-positive correlated to primary tumor (T). **(D)** ESTIMATE analysis shows risk score is positive-correlated to tumor purity (R=0.45). **(E)** ESTIMATE analysis shows risk score is negative-correlated to immune score in SKCM (R=-0.5). **(F)** ESTIMATE analysis shows risk score is negative-correlated to stromal score in SKCM (R=-0.27) .

To further investigate the prognosis potential of these prognosis-related PRRs in SKCM, we utilized and analyzed transcriptome data and clinical information of 468 SKCM cases from TCGA database. Through single factor (**Figure S3A**) and multiple factor test (21), we built a proportional hazards (Cox) model and 8 PRRs were selected in this Cox model, which were ZBP1, TLR7, NOD2, AIM2, TLR2, IFIH1, DDX60, and DDX58. According to risk score, 468 patients of SKCM were equally divided into low risk group and high risk group, and the expression of these 8 PRRs was decreasing with the increase of risk score (**Figure 2A**). Survival curve shows this prognosis model has better prognosis accuracy than any single PRR molecule (**Figures 2B**, **S3B**). To confirm the reliability of this PRR-based prognosis model, we calculated the ROC curve and results showed this prognosis model was credible (AUC=0.663) (**Figure S3C**). Next, we downloaded another cohort of melanoma from GEO database (GSE65904) to test the reliability of PRR-based prognostic model. And result shows this model also has value of predicting patients’ prognosis in this testing dataset (**Figure S4**).

To compare the clinically prognosis capability of this PRR-based prognosis model with other indicators, we performed univariate hazard ratio analysis to test the hazard ratio of individual age, patient gender, tumor stage, tumor size (T), lymph node spreading (N), metastasis (M) and PRR-based risk score in SKCM. We found PRR-based risk score had highest hazard ratio (p<0.001, HR=1.870) (**Figure S3D**), which means PRR-based risk score has more clinical value than other indexes. Next, utilizing multivariate Cox regression, we confirmed that risk score can serve as an independent prognostic predictor (p<0.001, HR=1.770) (**Figure S3E**). Furthermore, to analyze the relationship between risk score and tumor stage, we test the correlations between risk score, expression of PRR genes with TNM index, we found the size and extent of primary tumor (T) was highly positive-correlated with rick score (**Figure 2C**), and accordingly, negative-correlated with the expression of PRRs (**Figure S5**).

What’s more, we followed the interest in the relationship between tumor purity and risk score. Utilizing ESTIMATE algorithm, we analyzed the correlations between risk score and diverse ESTIMATE indexes. We found high risk score patients had higher tumor purity score, lower immune score, and lower stromal score than low risk score patients (**Figures 2D**–**F** and **Figure S6**), indicating intratumor immune microenvironment features are different between low risk score tumors and high risk score tumors, and high risk score tumors may have lower immunocytes’ infiltration.

### High Risk Score Indicates Low Immunocyte Infiltration and Weak Immune Activity in SKCM

**Figure 3:**
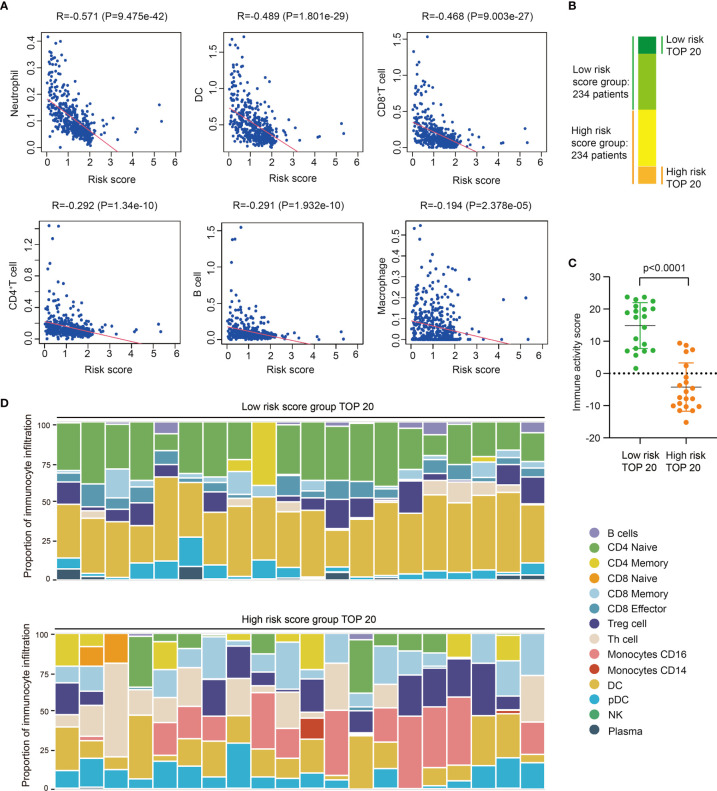
High risk score indicates low immunocyte infiltration and weak immune activity in SKCM. **(A)** Correlation analysis between risk score and different immunocyte infiltration, including Neutrophil, DC, CD8^+^T cell, CD4^+^T cell, B cell, and macrophage, and immunocyte infiltration data of different SKCM samples is downloaded from TIMER database. **(B)** The groups of SKCM samples used for immune activity and immune-infiltration analysis. **(C)** Immune activity of TOP20 low risk SKCM samples and TOP20 high risk SKCM samples in TIP analysis (t test, P<0.0001). **(D)** Immunocyte composition of TOP20 low risk SKCM samples and TOP20 high risk SKCM samples in TIP analysis.

Based on data of TIMER immune-gene database which collects immunocytes’ infiltration score of TCGA tumor samples, we further investigated the correlation between risk score and immunocyte infiltration, including neutrophils, dendritic cells (DC), macrophages, CD4^+^T cells, and CD8+T cells. Results shows risk score is negative-correlated with the infiltration of immunocytes in SKCM, especially neutrophils, DC, and CD8^+^T cells (**Figure 3A**), indicating these immunocytes could be much less infiltrated in tumors of high risk score group, which is consistent with the previously work revealing the diversity of tumor-infiltrating lymphocytes in melanoma (22).

Then we selected 20 highest risk score and 20 lowest risk score patients to further analyze their difference of immune activity and immunocyte composition (**Figure 3B**). Utilizing Tracking Tumor Immunophenotype (TIP) analysis, we found low risk score patients significantly have higher immune activity score than high risk patients (P<0.0001) (**Figures 3C**, **S7A**), and the expression of immune signature genes are also higher in low risk patients (**Figures S7B, C**). We also found the composition of infiltrated immunocytes are significantly different between low risk group and high risk group (**Figures 3D**, **S8**), showing there are less infiltrations of naïve CD4^+^ T cells and DCs, meanwhile more CD16^+^ monocyte and CD8^+^ T cells in tumors of high risk score group. Thus, we proposed these infiltrated immunocytes may play certain regulatory roles in the progress of SKCM, and this difference of immunocyte infiltration may be the reason of PRRs expression variation.

### Expression of PRRs Positive Correlated With That of Immune Checkpoint Molecules in SKCM

**Figure 4:**
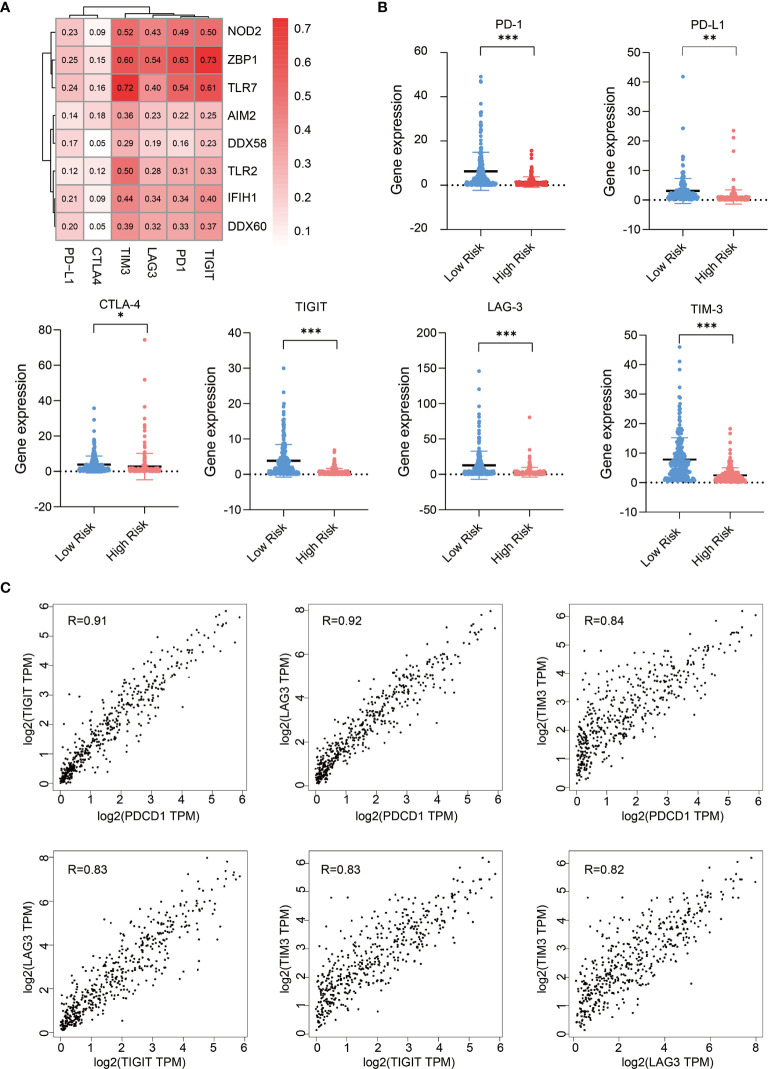
Expression of PRRs positively correlated with that of immune checkpoint molecules in SKCM. **(A)** Pearson correlation analysis reveals the correlations between 8 PRRs and immune checkpoints. **(B)** Expression of immune checkpoint genes in low risk and high risk SKCM patients (n=234 in low risk group, n=234 in high risk group, “*” means p<0.05, “**” means p<0.01, “***” means p<0.001). **(C)** The expression correlation analysis between each two of these immune checkpoint genes (PDCD1, LAG3, TIM3, and TIGIT).

Next, we analyzed the expression correlations between immune checkpoint molecules and PRR molecules in SKCM samples (23), these immune checkpoint molecules including PD-1, PD-L1, CTLA-4, LAG-3, TIM-3, and TIGIT. Pearson correlation coefficient shows the expression of immune checkpoints, especially TIM3, LAG3, TIGIT, and PD1, are highly positive-correlated with that of PRR molecules (**Figure 4A**). We also analyzed the expression of these immune checkpoint molecules in low risk and high risk tumor samples, we found high risk SKCM samples have lower expression abundance of these immune checkpoint molecules (P<0.05) (**Figure 4B**). This result suggests that high risk SKCM patients may be not sensitive to immune checkpoints therapy, as some PRR downstream effectors directly participates in or indirectly facilitates the process of tumor neoantigen presentation. Therefore, application of immune-checkpoint inhibitors in high risk patients need to be more carefully evaluated (24, 25).

Interestingly, as we found the correlation features of TIM3, LAG3, PD-1, and TIGIT with PRRs are similar in melanoma, we checked their correlation within each other, and found their expressions are also highly correlated with each other in melanoma (R>0.82) (**Figure 4C**), suggesting there are some molecular subsets of clinical relevance in SKCM which could be considered combinedly in medical research and clinical treatment.

### Expression of Immune Effector Genes Play a Positive Role in SKCM Prognosis

**Figure 5:**
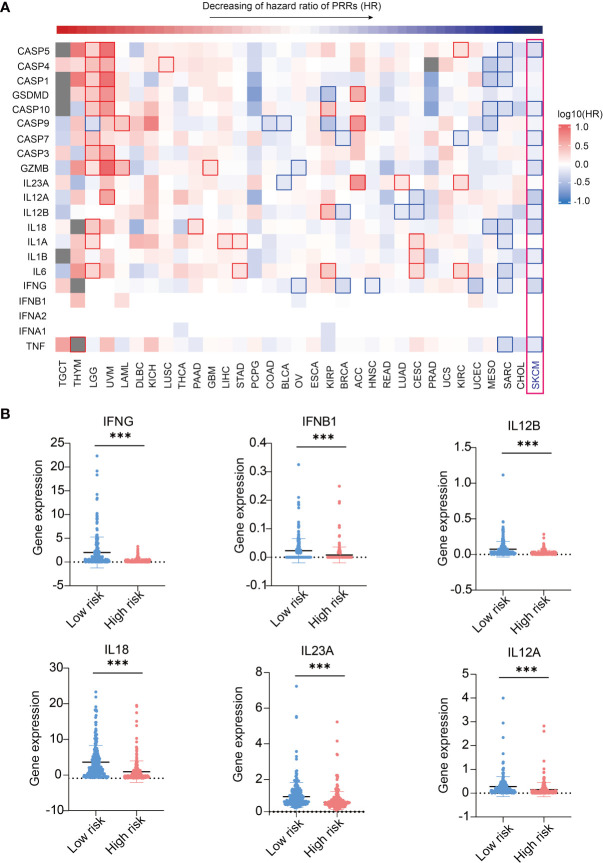
Expression of immune effector genes play a positive role in SKCM prognosis. **(A)** Pan-cancer prognosis analysis shows the expression of PRR-driven effector molecules highly correlated to SKCM prognosis (33 tumor types, 9502 samples). **(B)** 6 inflammatory cytokines express higher in low risk SKCM patients (n(low risk)=234, n(high risk)=234, “***”means p<0.001).

As we found expressions of PRRs were highly correlated to SKCM prognosis, we were interested in whether expression of PRRs’ downstream molecules, including innate immune signaling adaptors and immune effector molecules, could have similar correlations with survival times (26). We first analyzed the prognosis correlations of PRR downstream signaling molecules and found they are not correlated to prognosis in SKCM (**Figure S9** and **Table S2**), indicating PRRs’ prognosis potential in SKCM do not relate to the expression level of downstream immune signaling molecules. Then we analyzed the correlations of prognosis with the expression of effector molecules in innate immunity by GEPIA2 analysis. Interestingly, 11 out of 21 molecules are significantly positive correlated to prognosis in SKCM (**Figure 5A** and **Table S3**, and **Figure S10**). In addition, we found expression of 6 inflammatory cytokines, including IFNγ, IFNβ1, IL-12B, IL-12A, IL-18, and IL-23A are lower in high risk score SKCM samples(**Figure 5B**). These results indicating the expression of effector genes may play a critical positive role in SKCM prognosis along with upstream PRR molecules.

### Single Cell Sequencing Analysis Reveals PRRs’ Specific Expression on Macrophages in Melanoma

**Figure 6:**
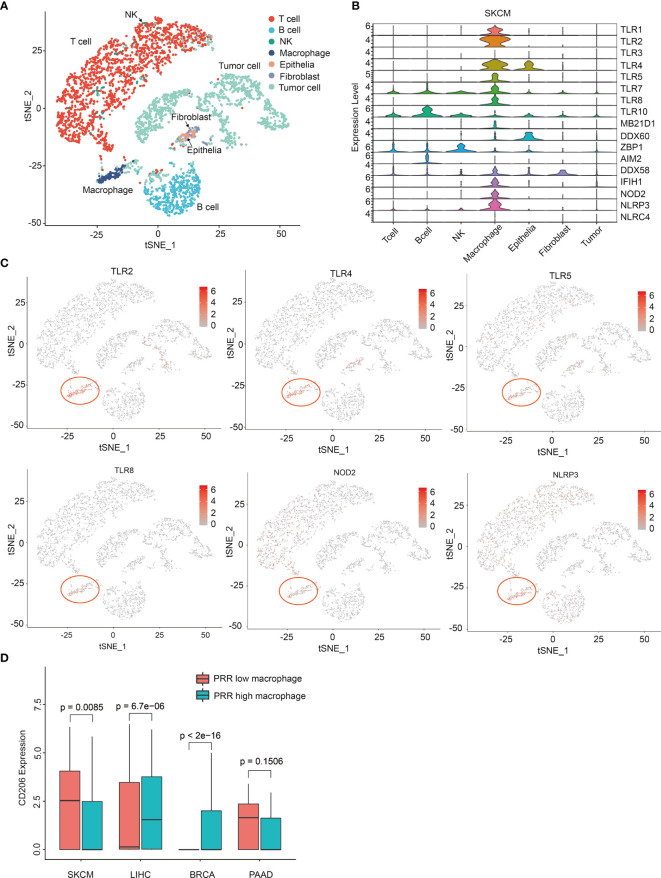
Single cell sequencing analysis reveals PRRs’ specific expression on macrophages in melanoma. **(A)** Single-cell expression profiles distinguish different cell types in SKCM (tSNE). **(B)** Single-cell PRR expression profiles of different cellular types in SKCM. **(C)** tSNE graphs show PRRs’ specifically express on macrophages in SKCM samples. **(D)** CD206 has a specific low expression in PRR high macrophages only in SKCM samples (P<0.01).

Next, we tried to explore the underlying mechanism of PRRs highly correlated with prognosis specially in SKCM, and we thought the cells that express PRRs are different in SKCM from that in other tumors. So, we investigated the expression of PRRs in different cells from SKCM and other tumors. Using single cell sequencing data of melanoma from GEO database (GSE72056), we classified 4645 cells as 7 cell subpopulations, including T cell, B cell, NK, macrophage, epithelia, fibroblast, and tumor (**Figure 6A**). Interestingly, we found the expression of PRRs family were concentrated significantly in macrophages in SKCM (**Figures 6B, C**), while PRRs are not significantly highly expressed in macrophages from other types of tumors, including breast invasive carcinoma (BRCA), liver hepatocellular carcinoma (LIHC), and PAAD (**Table S4** and **Figure S11**). These results suggest that PRR-high expressing macrophages might specifically infiltrate in melanoma and be beneficial to prognosis.

### Infiltration of CD206 Low Macrophages in Tumor Could Beneficial to SKCM Prognosis

As the infiltration of total macrophages was low-correlated to risk score in SKCM (Cor = -0.194) (**Figure 3A**), we thought it must be some subgroup of macrophage that be critical for SKCM prognosis, which should be PRR-high macrophages rather than other groups of macrophages. So, we tried to identify some genetic feature between PRR-low and PRR-high macrophages. According to cellular risk scores, we divided these macrophages into two groups and then performed enrichment analysis. Interestingly, we found the expression of CD206 was significantly lower in PRR-high macrophages than that in PRR-low from melanoma, while is not the case in other types of tumors (**Figure 6D**).

As several studies suggested that CD206 could be a candidate marker of M2 macrophage, which usually played the role of promoting tumor growth in intratumor microenvironments (27, 28). So, we next analyzed the expression of CD163, a more classical M2 macrophage marker to further verify the feature of CD206 high and CD206 low macrophages. Result showed there was no significant difference between the expression of CD163 in low risk score and high risk score group of macrophages (p=0.25) (**Figure S15B**), suggesting that the characteristic of these high risk score macrophages in melanoma may be not fully same as M2 macrophage subpopulation (29). These data indicates that the infiltration of CD206-low and PRR-high macrophages may be beneficial to the prognosis of melanoma. To investigate the intracellular pathway features between PRR low and PRR high macrophages, utilizing GO analysis, we found the expression of autophagy and protein polyubiquitination genes are specific down-regulated in PRR high macrophages, and the expression of neutrophil degranulation genes, viral gene expression, and NF-KB genes are specific up-regulated in PRR high macrophages (**Figure S16**). Utilizing KEGG analysis, we found the expression of ubiquitin mediated proteolysis, NOD-like receptor, TLR signaling, valine, leucine, and isoleucine pathways are specific down-regulated in PRR high macrophages, and the expression of protein processing, thyroid hormone, amino sugar, nucleotide sugar, AMPK, TNF, FOXO, and insulin signaling pathways are specific up-regulated in PRR high macrophages (**Figure S17**). These results show that the intracellular pathway signature of these two groups of melanoma-infiltrated macrophages were significantly different, and PRR high macrophages have the feature of stronger effective immunity (30). However, molecular mechanisms of these two types of macrophages function in melanoma need to be further studied to specifically clarify.

### PRRs Specifically Express on Macrophages in Mice Melanoma Samples

**Figure 7:**
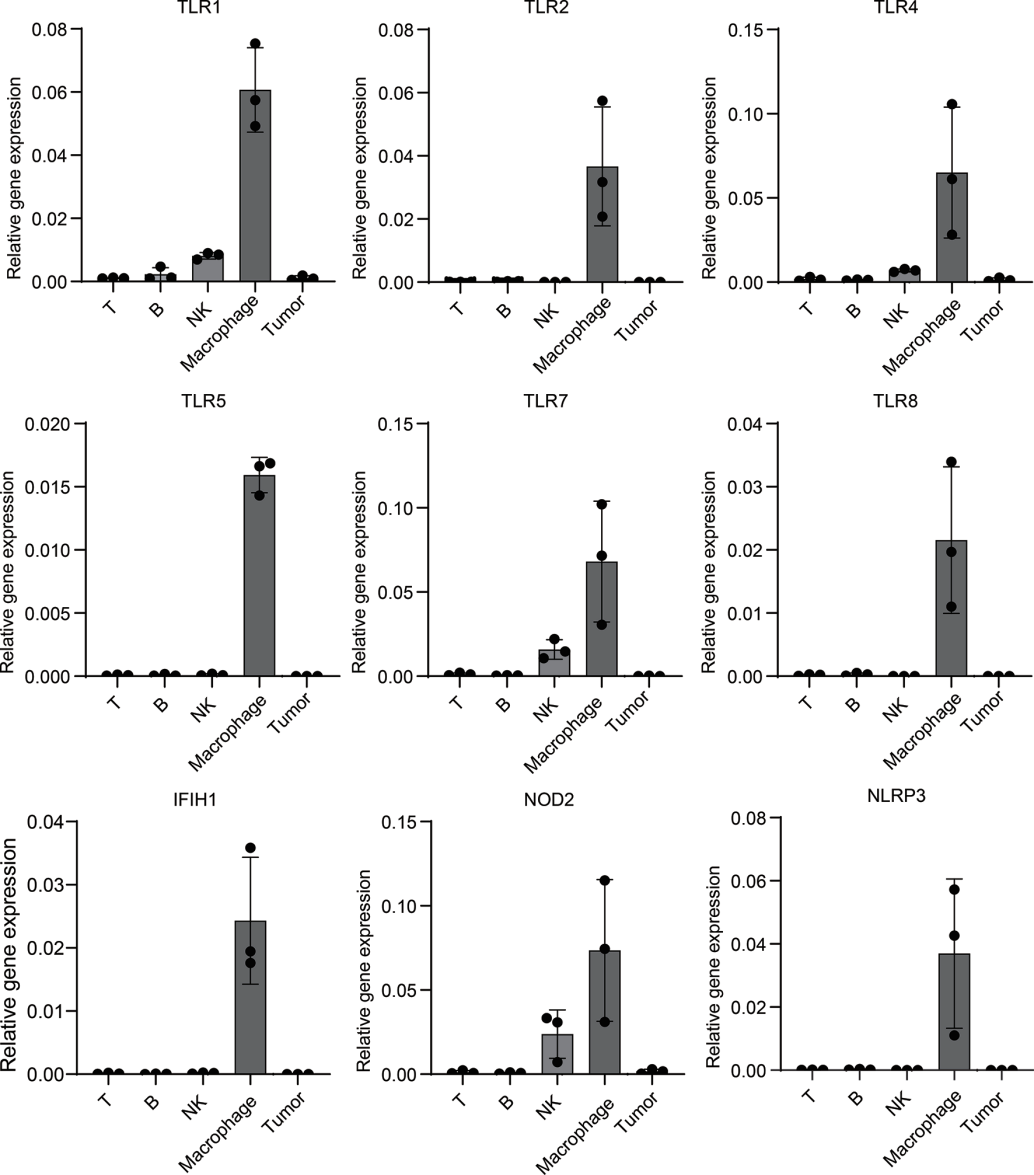
Mouse model experiment shows PRRs’ specific expression on macrophages in melanoma samples. Melanoma cell line B16 cells were injected into subcutaneous of C57/B6-L mice (n=3), after injection of 18 days, different types of immunocytes were isolated, and then total RNA were extracted. After RT-PCR, qPCR analysis was performed to detect the expression of PRRs. The relative expression of PRRs were normalized to that of GAPDH. Results were analyzed in GraphPad Prism version 8.0.1.

To further verify PRRs’ specific expression on macrophages in melanoma, we build a mouse melanoma model by subcutaneous injection of B16 cell. Next, we isolated tumor-infiltrated immunocytes including T cells, B cells, NK cells and macrophages from tumors, qPCR experiment shows PRRs indeed highly expressed in macrophages rather than other types of immunocytes and tumor cells (**Figure 7**), and these results are highly consistent with the single-cell sequencing results of PRRs’ expression.

## Discussion

PRR family is one of the most important functional families in innate immunocytes that plays diverse roles in multiple diseases. In our study, analysis of the prognosis correlations of PRRs in pan-cancer scope enabled us to determine their specifically positive prognosis potentials in SKCM, and we found PRRs-based prognosis model have better prognosis value than any single PRR molecule. In addition, we found there are lower immune-infiltration and immune activity in patients with high risk score, meanwhile the composition of immunocytes were different between high and low risk score group. Next, we found the expression of immune-checkpoint molecules in SKCM patients were positively correlated with risk score, which could be immune therapeutic indicator in future. We also found that the expression of effector molecules downstream of PRRs including several cytokines may be beneficial to SKCM prognosis. Moreover, we analysed the single-cell sequencing data of several cancers, results suggested that the expression of PRRs are specific high in a subpopulation of macrophages from melanoma samples, and these PRR high macrophages are shown as the characteristic of CD206 low expression. Finally, we build a mouse melanoma model to verify PRRs’ specific expression on macrophages, and results shows PRRs indeed highly expressed on macrophages in melanoma samples, that means the infiltration of PRR high macrophages may be the reason of why PRRs have positive prognosis potentials in SKCM.

Another interested question of pan-cancer survival analysis is why the expression of the whole PRR family specifically suggests poor-prognosis in LGG patients that contrary to the result of SKCM. Inflammation promotes LGG progression within the special environment of the central nervous system may be possible explanations. A series of study have explained that the formation of inflammation microenvironment in glioma promotes immunosuppressive, and up-regulate of inflammation with followed cell damage may boost the increase of the glioma’s degree of malignancy, meanwhile impair the function of brain tissue especially nerve cells and cause worse prognosis (31). In terms of molecular mechanism, the secretion of inflammatory cytokines promotes the expression of granulocyte-macrophage colony-stimulating factor (GM-CSF), thereby enhancing the recruitment of microglia/macrophages and microglia-dependent glioma invasion (32), and inhibition of CSF-1R can alter macrophage polarization and block glioma progression (33). Nevertheless, the role of PRR family suggesting specific poor-prognosis in LGG still needs to be further clarified (34).

Several studies and clinical trials have found that the activation of PRR is conducive to the therapeutic effect of immune checkpoint inhibitors in melanoma (35). For instance, the application of TLR1/2 agonists (Diprovocim) can effectively enhance the therapeutic effect of PD-1 immunotherapy and prolong the survival time of mice in melanoma (36). Moreover, CMP-001, a CpG DNA TLR9 agonist, was reported to have the ability to activate tumor-associated plasmacytoid dendritic cells to produce interferon, which in turn induces antitumor systemic immunity (37). In 2020, FDA has approved CMP-001 in combination with nivolumab (Opdivo) and ipilimumab (Yervoy) as a treatment plan for patients with late-stage melanoma, and clinical trials are underway at the stage of II to determine confirmed objective response with CMP-001 in combination with nivolumab in subjects with refractory unresectable or metastatic melanoma (NCT04698187) (38). These studies have illustrated the close relationship between PRR and immune checkpoint immunotherapy in SKCM, and we believe that the activation of PRRs especially in macrophage could beneficial to SKCM patient’s immunotherapy (39).

As many studies have already revealed, the precious roles of PRR-induced immune responses and inflammations in tumorigenesis and antitumor activity are just like a double-edged sword (40). PRRs plays different roles in different types of cancers and even in different stage of one cancer, so it is not easy to explain their exact functions clearly with one molecule or pathway, which needs to be comprehensively evaluated in vast number of clinical samples. Thus, it is important and necessary to systematically evaluate the role of PRRs and their downstream responses in different types of clinical tumors, meanwhile considering the heterogeneity of cancer which cannot be ignored. Furthermore, as we found PRRs was specific expression on macrophages in melanoma samples, and expression of PRRs were specifically high-correlated to patient’s survival in SKCM, this result indicated that the functions of PRRs molecules in innate immunocytes, cancer-associated fibroblasts (CAF), and tumor cells are obviously different (41), so the role of PRRs family in different cells need to be discriminated and investigated separately. What’s more, expression of PRRs in macrophages could beneficial to antigen presentation and recruitment of immunocytes in tumor microenvironments, thereby enhancing the activation of adaptive immunity. These mechanisms may be also possible reasons for infiltration of PRR high macrophages indicating positive prognosis in melanoma (42, 43).

Overall, we found PRRs could be potential indicators for positive prognosis and immune-infiltration feature in SKCM, and the infiltration of PRR high macrophages may be the reason for PRRs’ positive prognosis correlation, leaving the underlying molecular mechanism to be resolved. Our findings have important implications for deepening the understanding of the different roles of PRRs in diverse tumor types. In summary, we demonstrated that PRR family have prognosis potential in SKCM, and PRR-base risk score will provide an alternative indicator for clinical prognosis and therapeutic strategies in melanoma.

## Data Availability Statement

The datasets presented in this study can be found in online repositories. The names of the repository/repositories and accession number(s) can be found in the article/**Supplementary Material**.

## Ethics Statement

The animal study was reviewed and approved by Ethics committee of Capital Medical University.

## Author Contributions

QZ and TW performed the bioinformatic analysis of pan-cancer data and single cell sequencing data from TCGA and GEO database. QZ and QW performed the mouse model experiments and the detection of PRRs in different cells. QZ, QW, TW, JX, TL, QL, QY, and PW analysed and interpreted the results. QZ, QY, and PW wrote the paper. All authors contributed to the article and approved the submitted version.

## Funding

This work was supported by grants from the National Natural Science Foundation of China (81971499, 82070765). Funding for open access charge was from National Natural Science Foundation of China.

## Conflict of Interest

The authors declare that the research was conducted in the absence of any commercial or financial relationships that could be construed as a potential conflict of interest.

## Publisher’s Note

All claims expressed in this article are solely those of the authors and do not necessarily represent those of their affiliated organizations, or those of the publisher, the editors and the reviewers. Any product that may be evaluated in this article, or claim that may be made by its manufacturer, is not guaranteed or endorsed by the publisher.
