# Supplementary material for: Pattern Recognition Receptors (PRRs) in Macrophages Possess Prognosis and Immunotherapy Potential for Melanoma
Source: Front Immunol. 2021 Nov 9;12:765615. doi: 10.3389/fimmu.2021.765615 (PMC8630683; doi:10.3389/fimmu.2021.765615)
Supplement: Supplementary file 1 [file DataSheet_1.docx]

Supplementary Figures

**Pattern Recognition Receptors (PRRs) in Macrophages Possess Prognosis and Immunotherapy Potential for Melanoma**


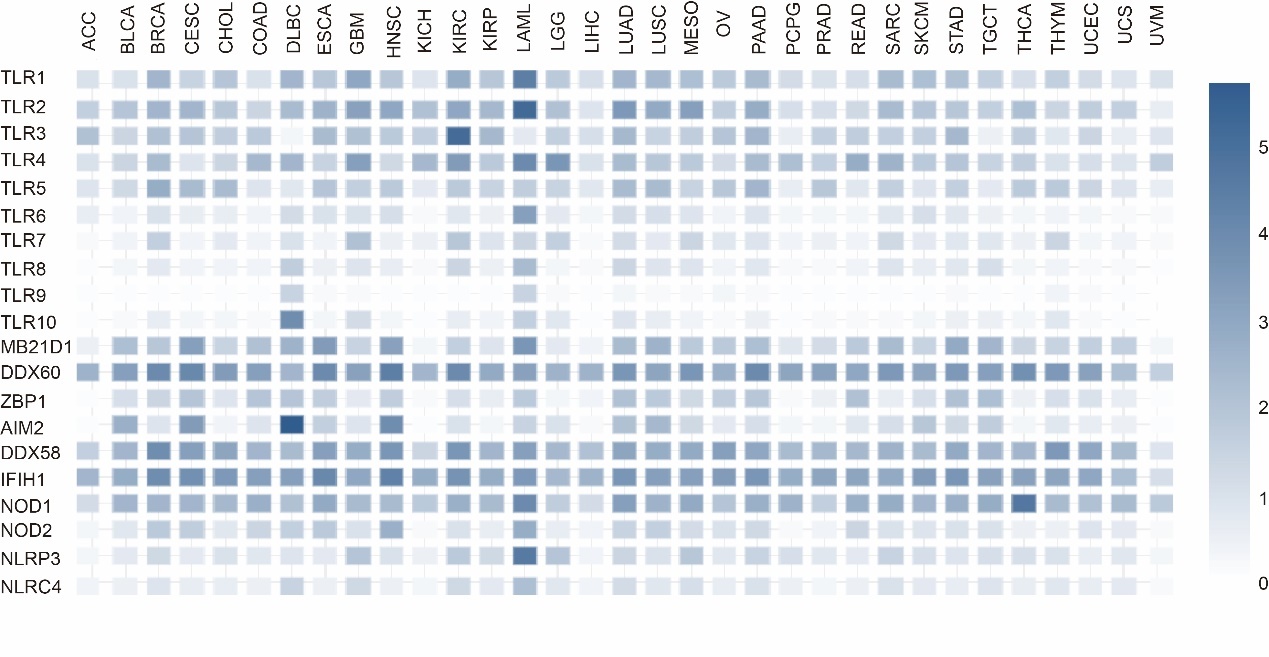


**Figure S1** The expression profiles of PRRs in 33 types of tumors.


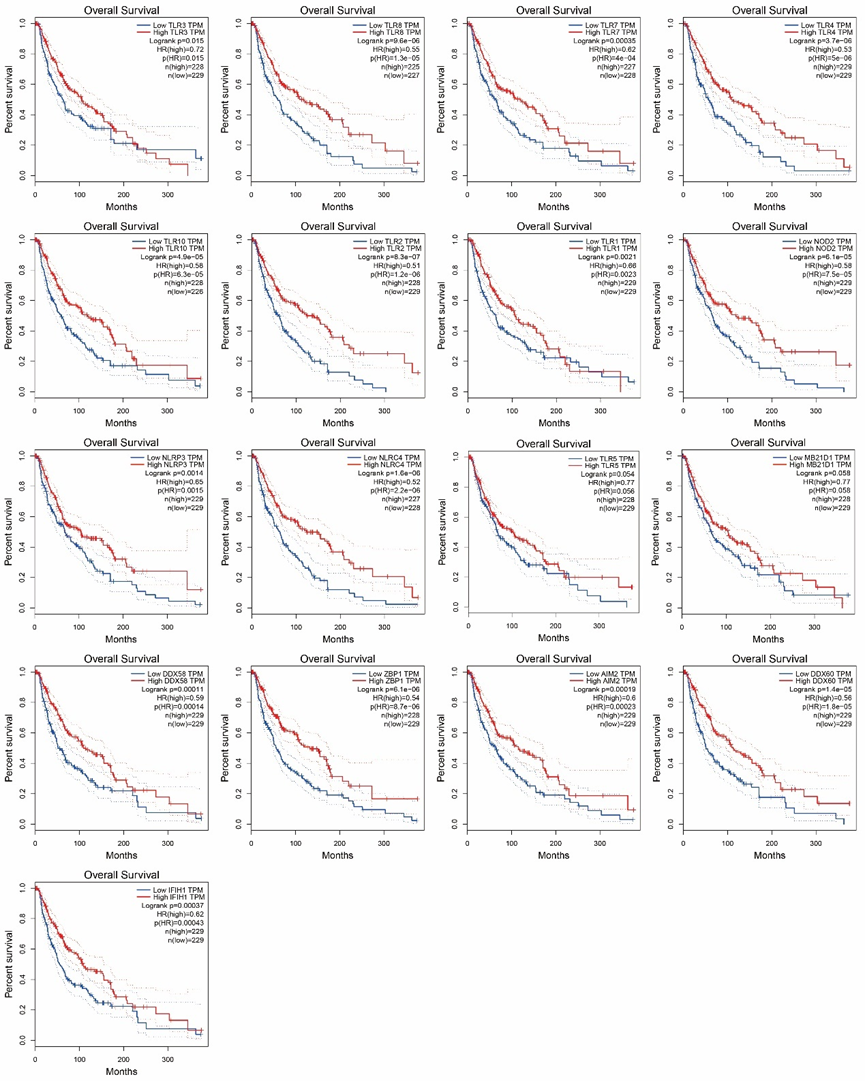


**Figure S2** The survival curves of 17 SKCM-prognosis-related PRR genes (pHR<0.05).


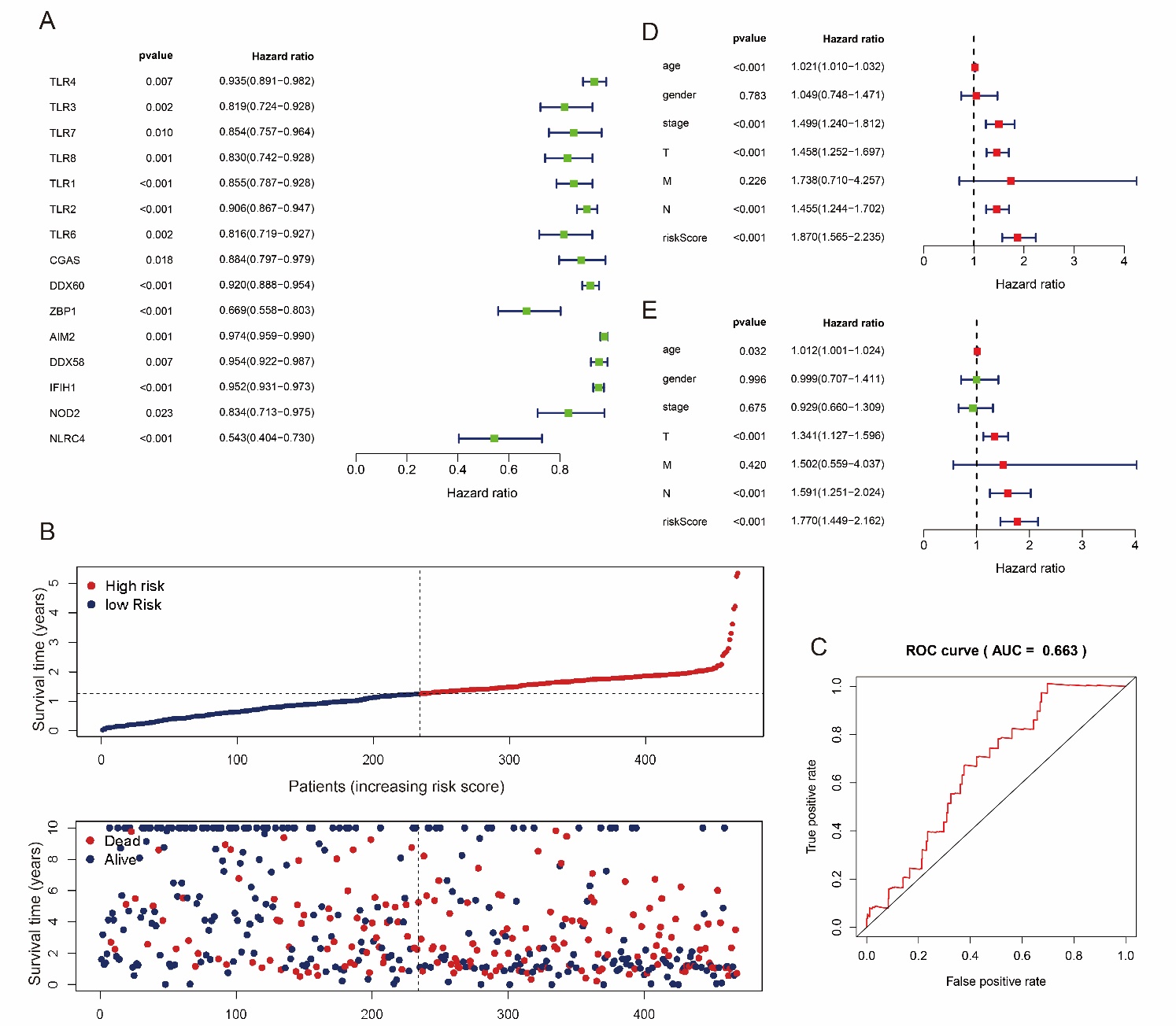


Figure S3 The construction of PRR-based COX prognosis model. **(A)** Hazard ratio of 15 PRRs in SKCM calculated by COX analysis. **(B)** Distribution of patients’ risk score and survival time, and status of SKCM (Alive/Dead). The black dotted line is the optimal cut-of value for dividing patients into low risk and high risk groups. **(C)** ROC curve of PRR-based COX prognosis model (AUC=0.663). **(D)** Univariate hazard ratio analysis of age, gender, stage, T, M, N, and risk score. **(E)** risk score (p < 0.001, HR = 1.770,) was presented as an independent prognostic predictor by multivariate Cox regression.


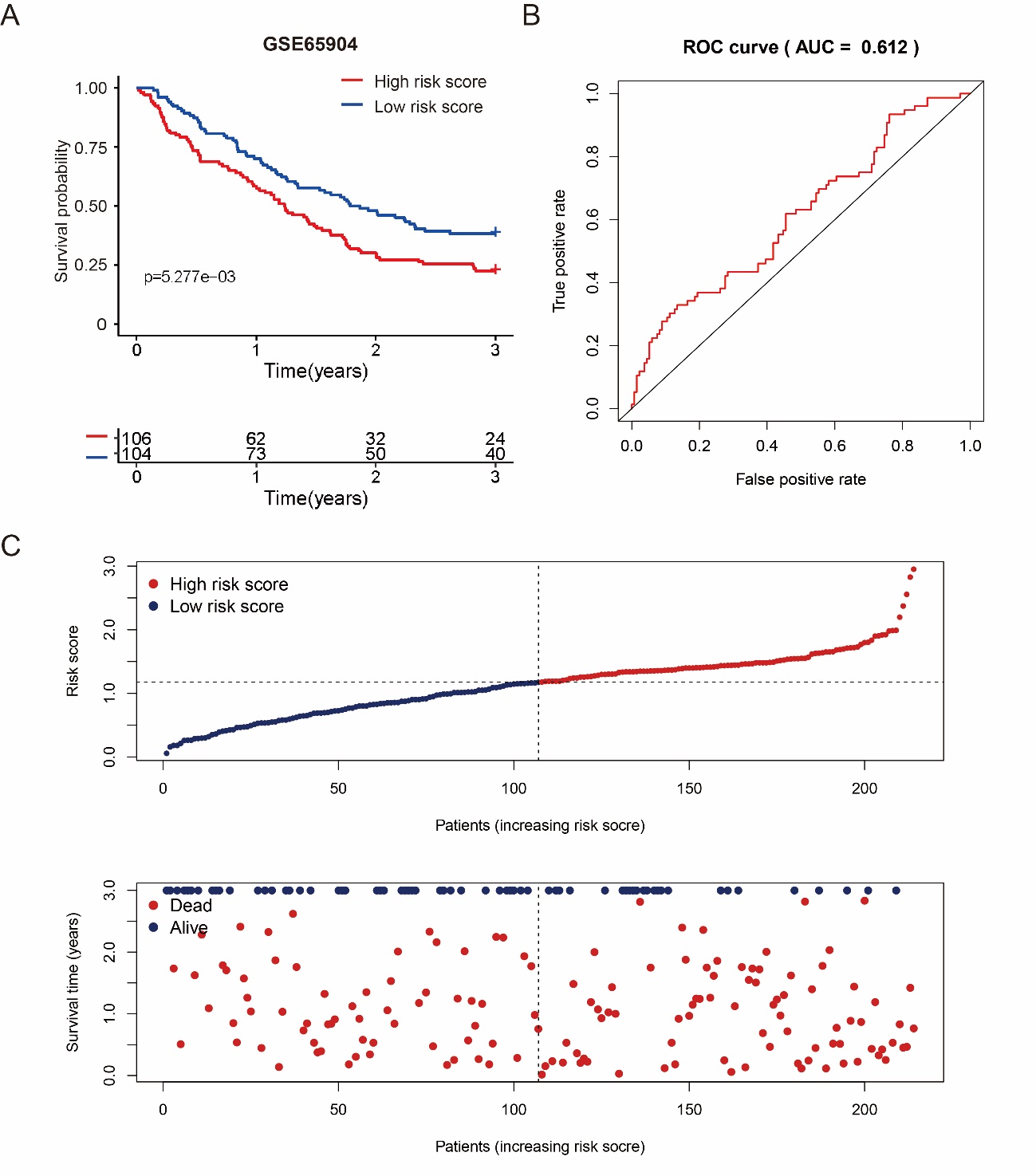


Figure S4 The verification of PRR-based COX prognosis model by analyzing testing dataset. **(A)** Kaplan-Meier curve of low risk score group and high risk score group of testing dataset (GSE65904), and samples which are no data of survival times are filtered. **(B)** ROC curve of testing dataset in PRR-based COX prognosis model (AUC=0.612). **(C)** Distribution of patients’ risk score and survival time, and status of testing dataset (Alive/Dead). The black dotted line is the optimal cut-of value for dividing patients into low risk and high risk groups.


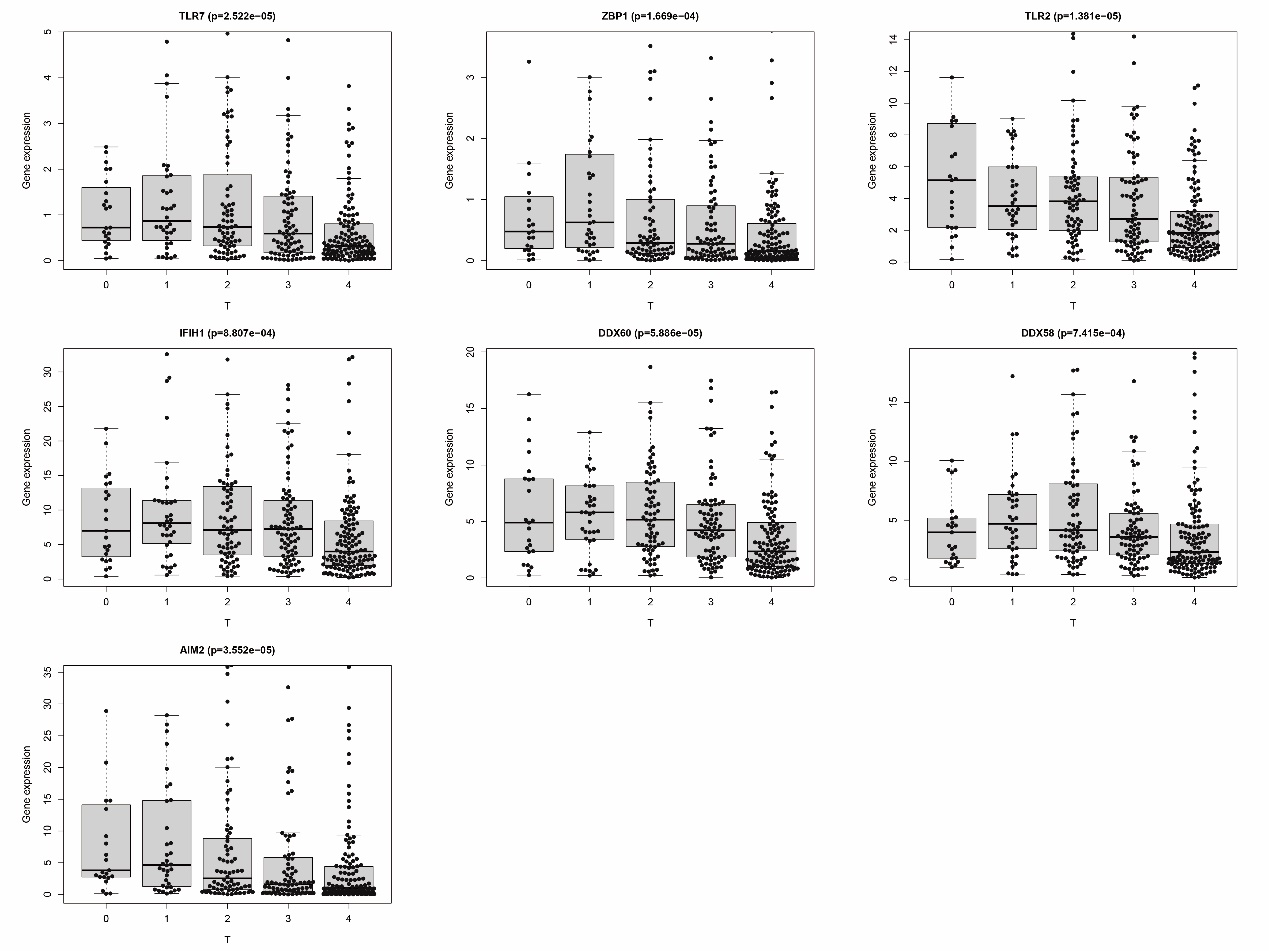


**Figure S5.** The expression of 7 PRR genes from COX model is high-correlated to tumor size (T)(P<0.05).


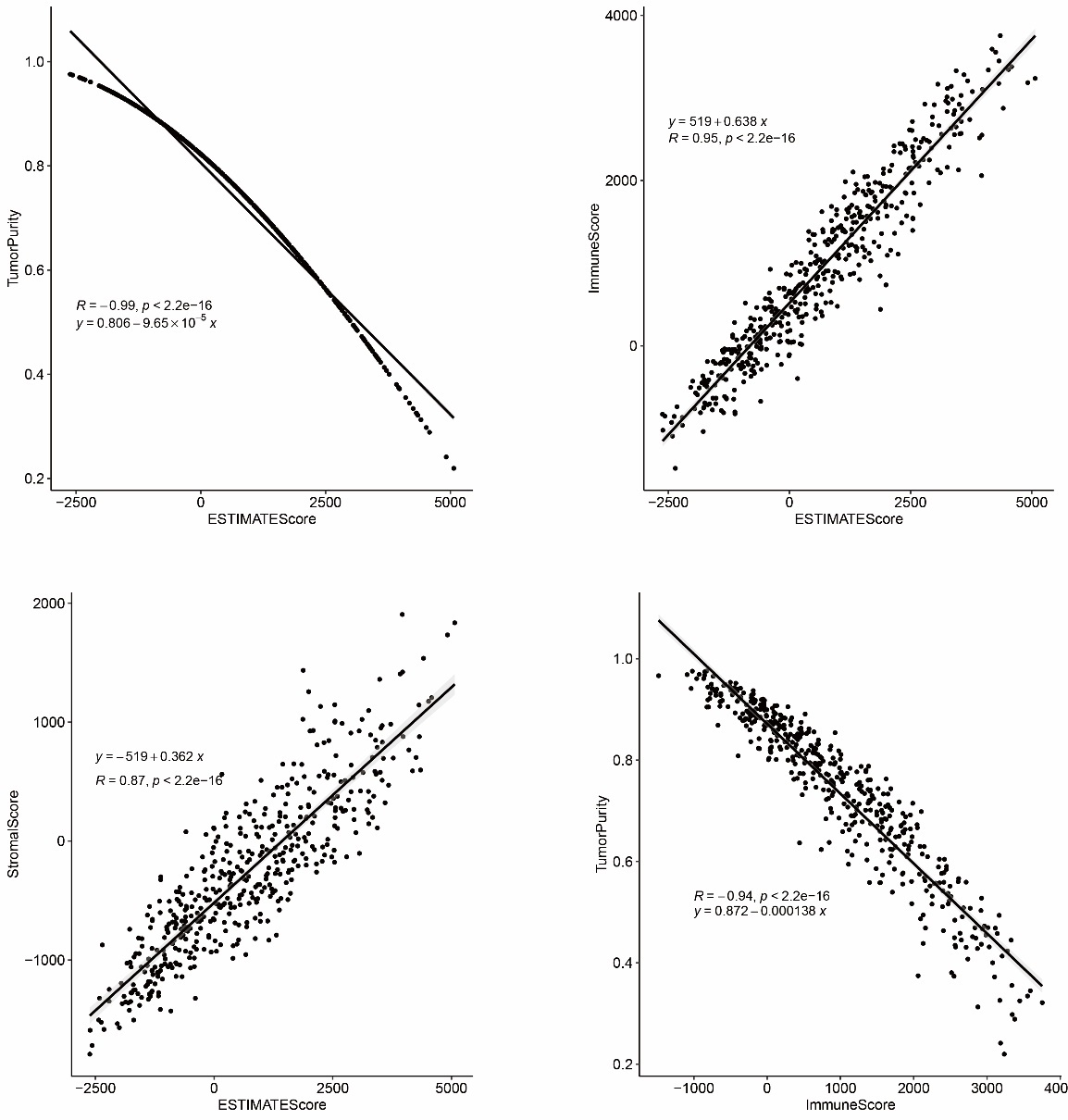


**Figure S6** ESTIMATE analysis of SKCM samples.


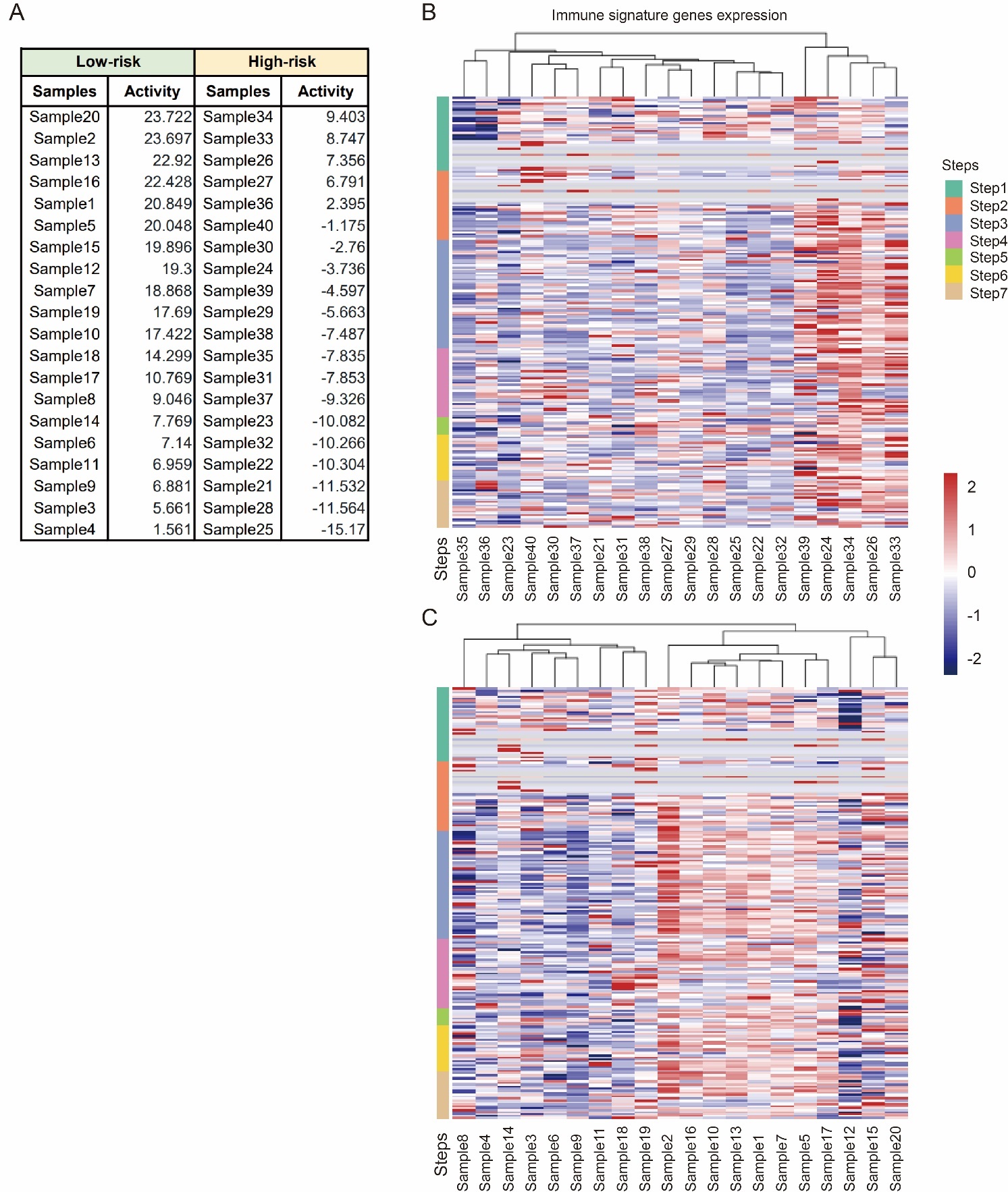


Figure S7 Immune activity and immune signature gene expression analysis by TIP. **(A)** The Immune activity score of top20 low risk score SKCM samples and top20 high risk score SKCM samples. **(B)** Heatmap shows immune signature gene expression of TOP-20 high risk score SKCM samples. **(C)** Heatmap shows immune signature gene expression of TOP-20 low risk score SKCM samples.


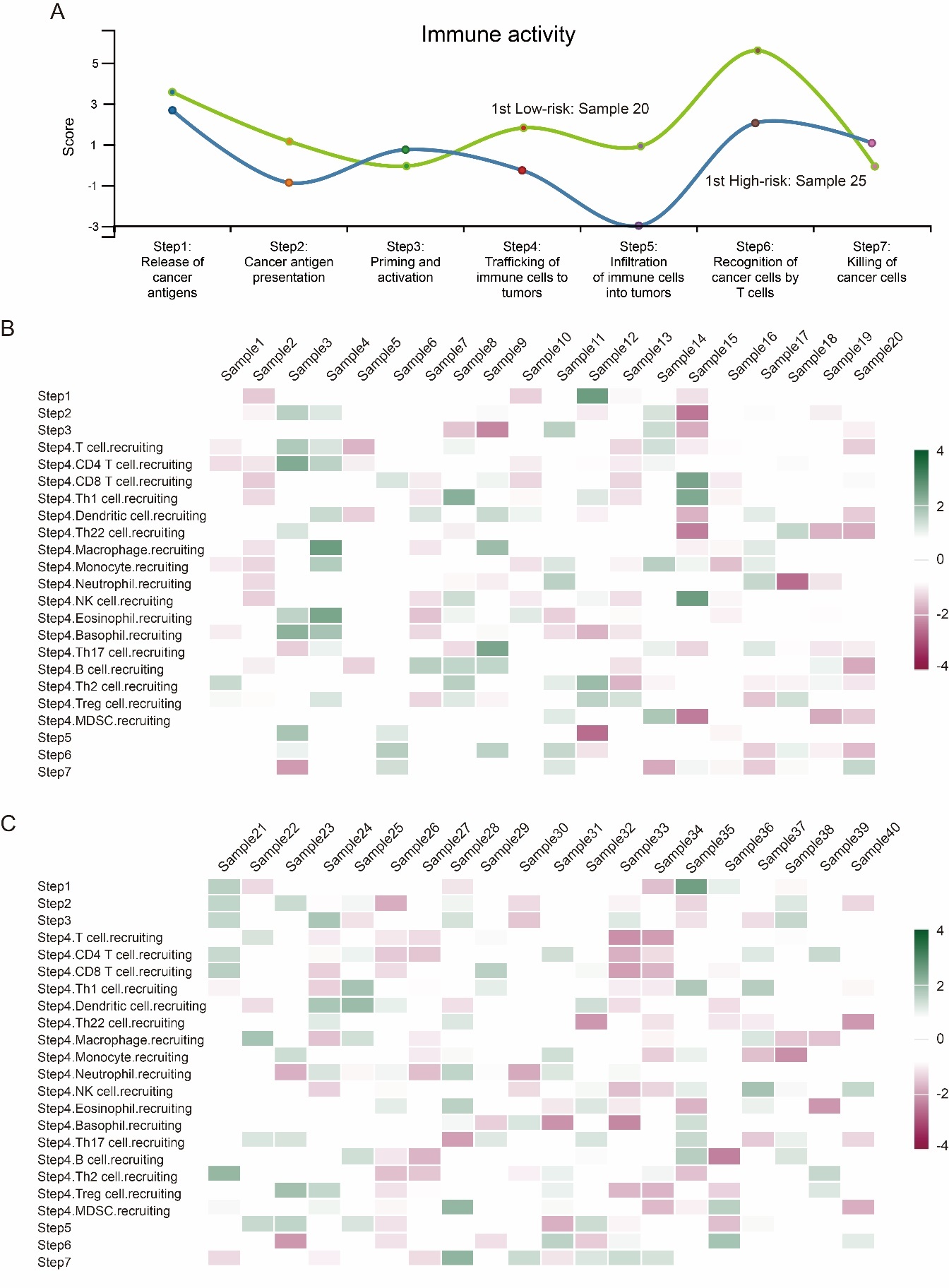


Figure S8 Immune activity analysis by TIP. **(A)** Schematic diagram of the step of TIP analysis. **(B)** The heatmap of score of diverse steps within top20 low risk score SKCM samples. **(C)** The heatmap of score of diverse steps within top20 high risk score SKCM samples**.**

**
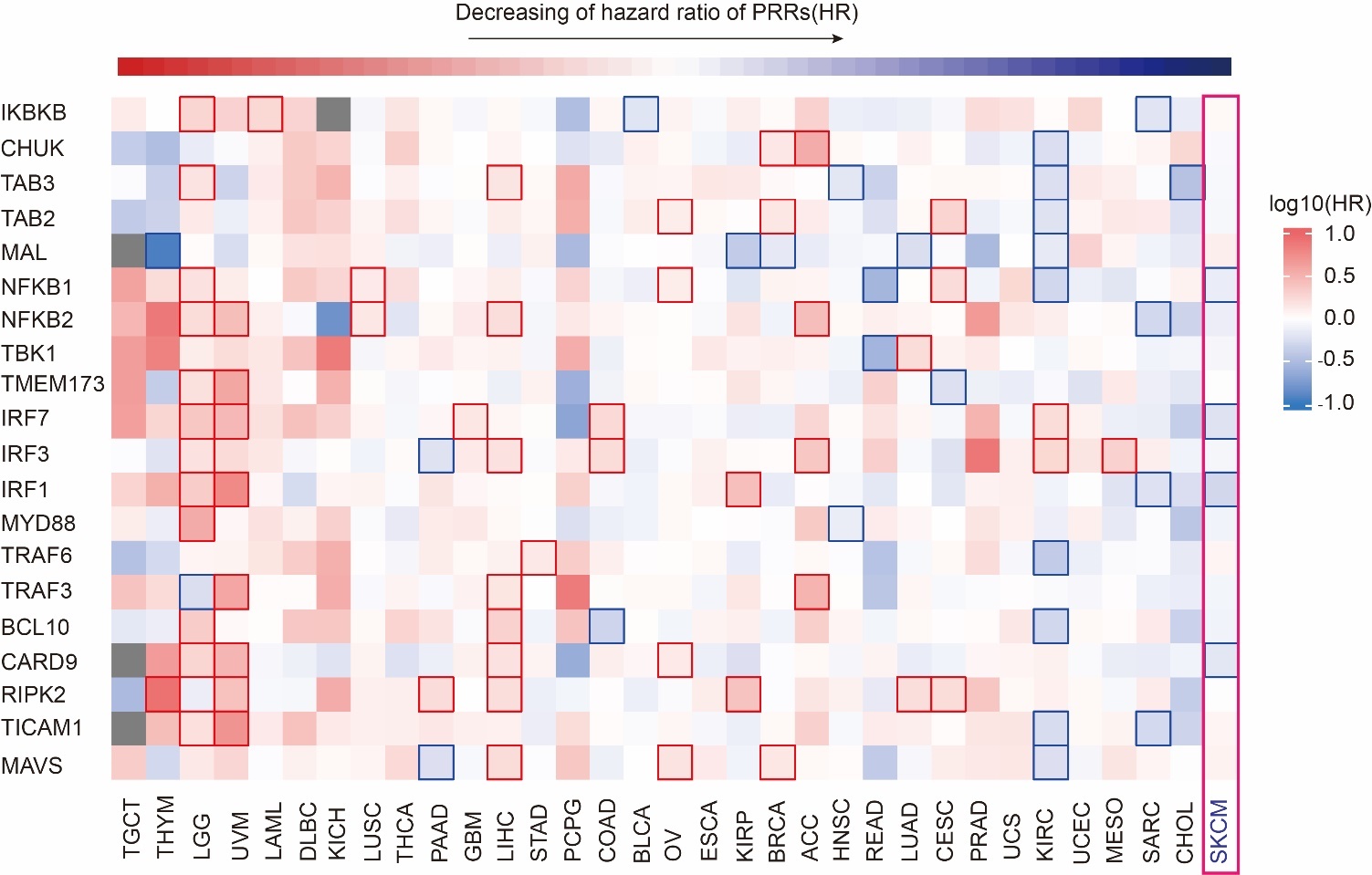
**

**Figure S9** Expression of innate immune-signaling molecules hardly correlated to SKCM prognosis (tumor types=33, samples=9502).


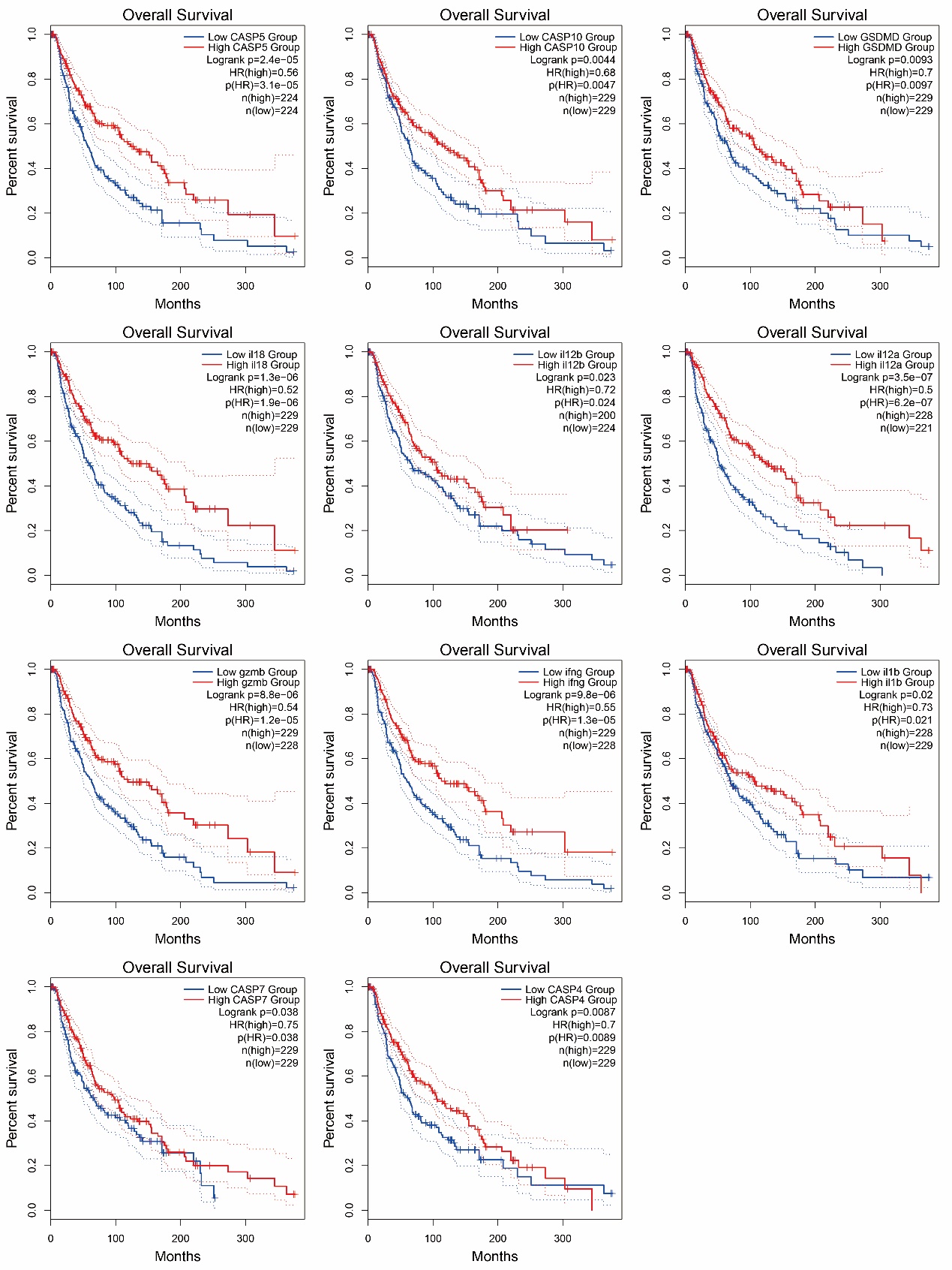


**Figure S10** The survival curves of 11 prognosis-positive effector molecules of innate immunity in SKCM (pHR<0.05).


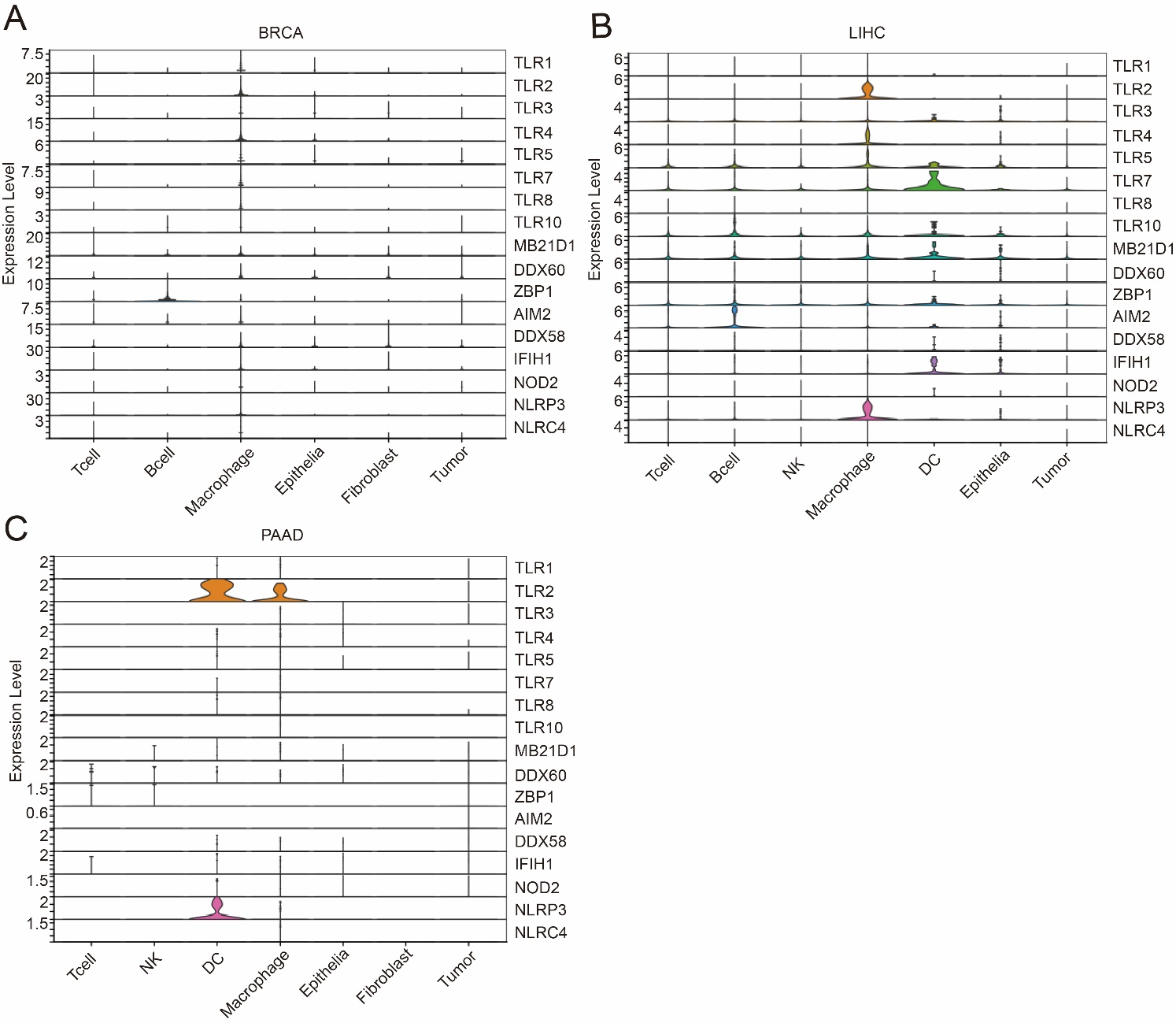


**Figure S11** Single-cell sequencing analysis reveals the expression of PRRs on different cell types in different tumors. **(A)** Single-cell PRR expression profiles of different cellular types in BRCA. **(B)** Single-cell PRR expression profiles of different cellular types in LIHC. **(C)** Single-cell PRR expression profiles of different cellular types in PAAD.


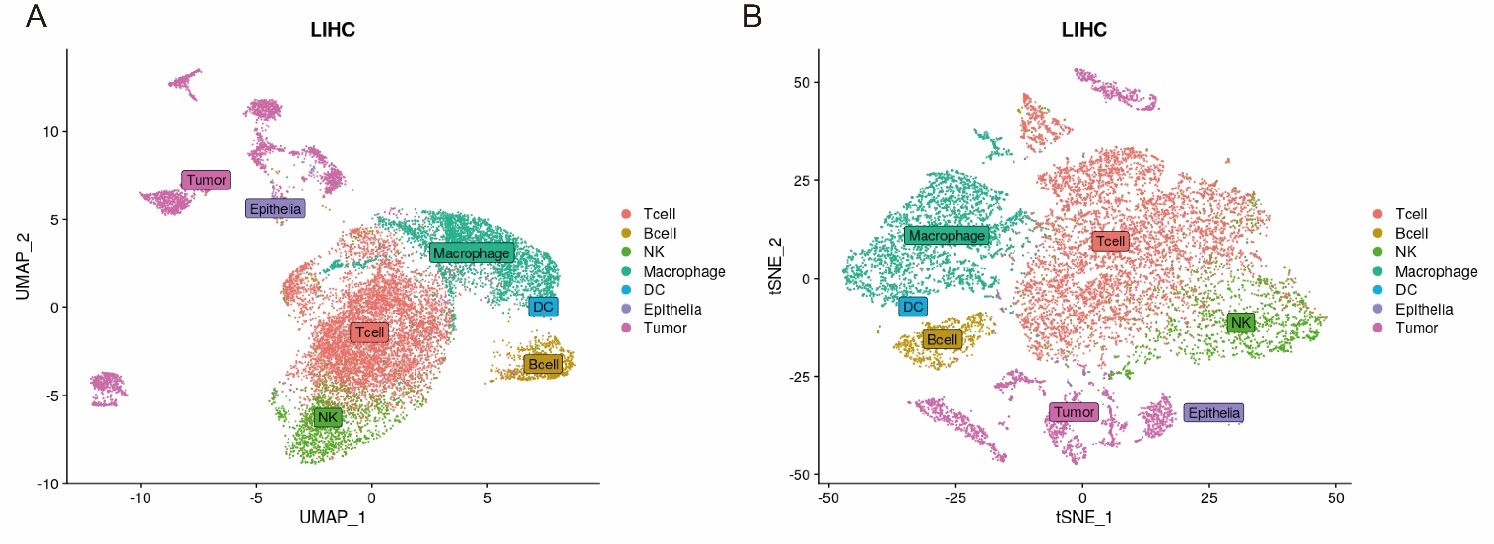


**Figure S12** Single-cell profiles of cell subpopulations and expression of PRRs in LIHC samples. **(A-B)** Single-cell expression profiles distinguish different cell types in LIHC.


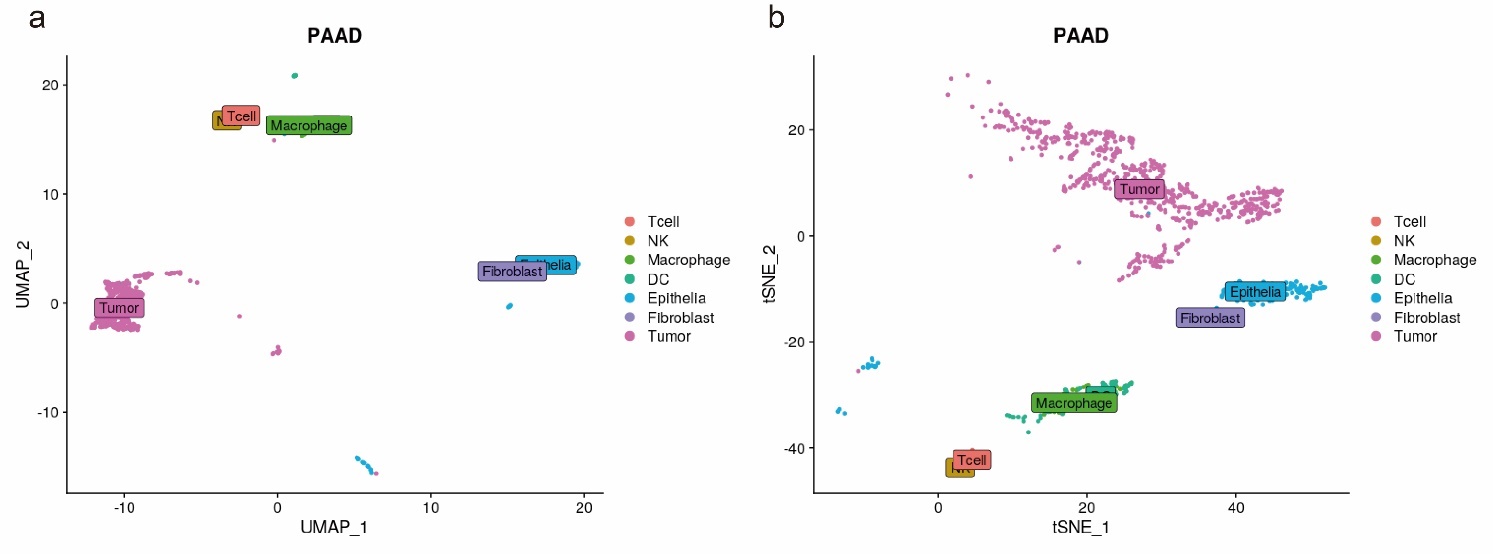


**Fig S13** Single-cell profiles of cell subpopulations and expression of PRRs in PAAD samples. **(A-B)** Single-cell expression profiles distinguish different cell types in PAAD.


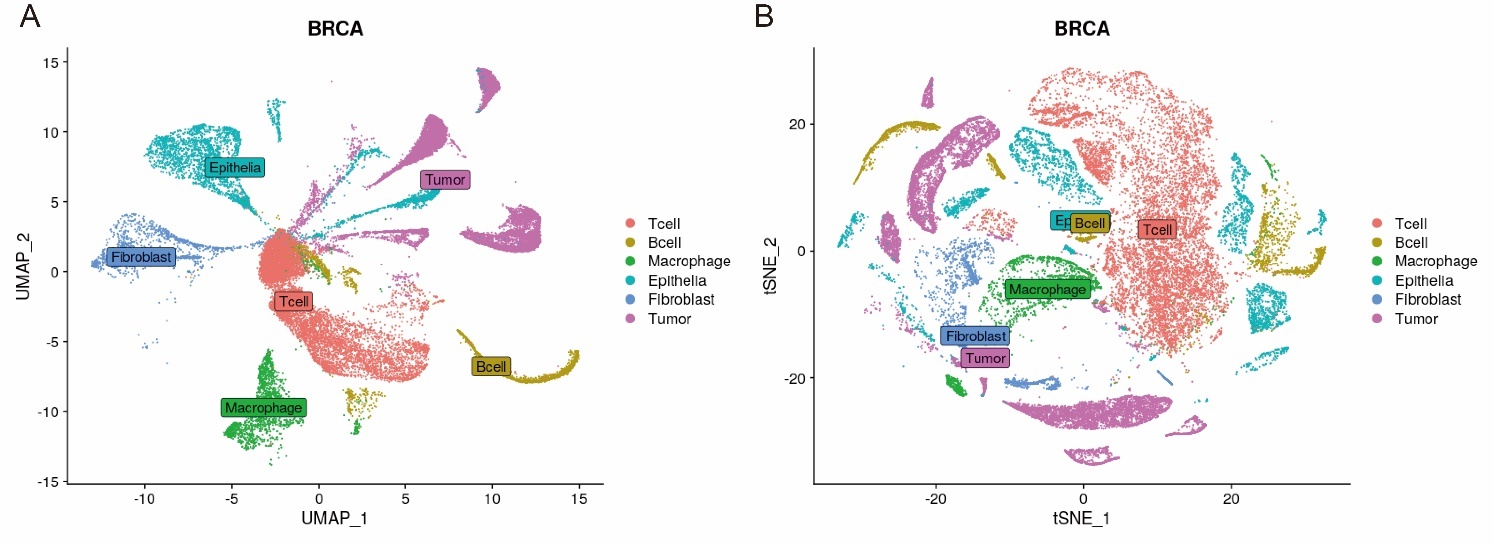


# Figure S14 Single-cell profiles of cell subpopulations and expression of PRRs in BRCA samples. (A-B) Single-cell expression profiles distinguish different cell types in BRCA.


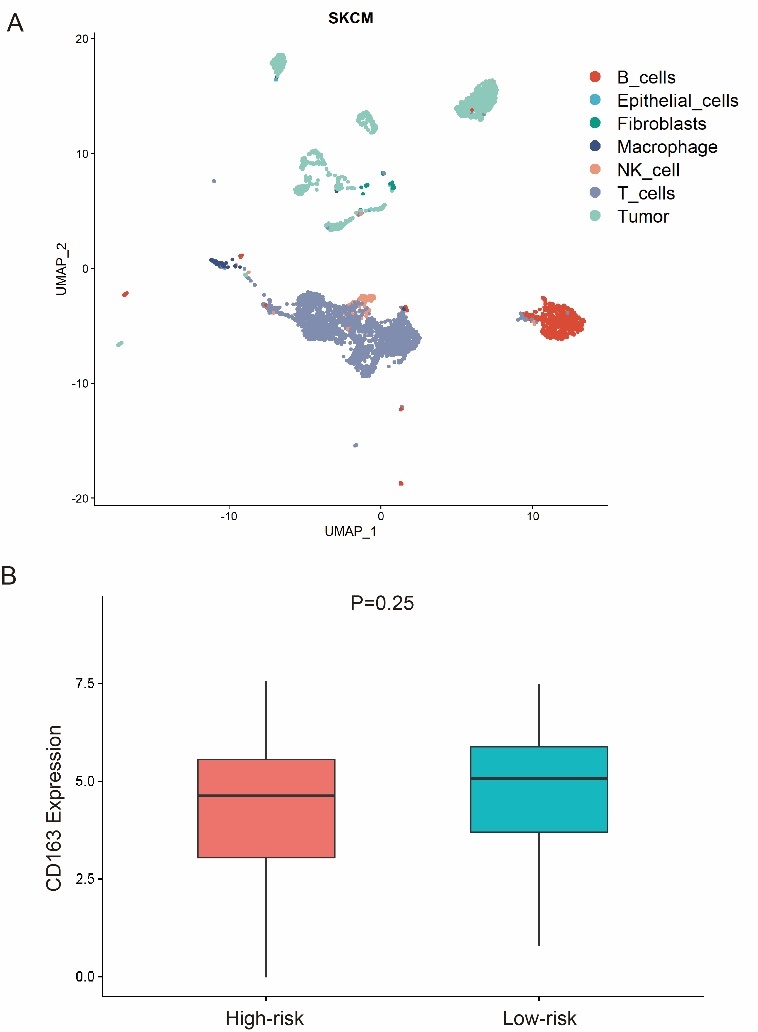


**Figure S15** Single-cell profiles of cell subpopulations and expression of CD163 in SKCM samples. **(A)** Single-cell expression profiles distinguish different cell types in melanoma（UMAP）. **(B)** CD163 is not significant expression in high-risk macrophages within SKCM samples (P=0.25).


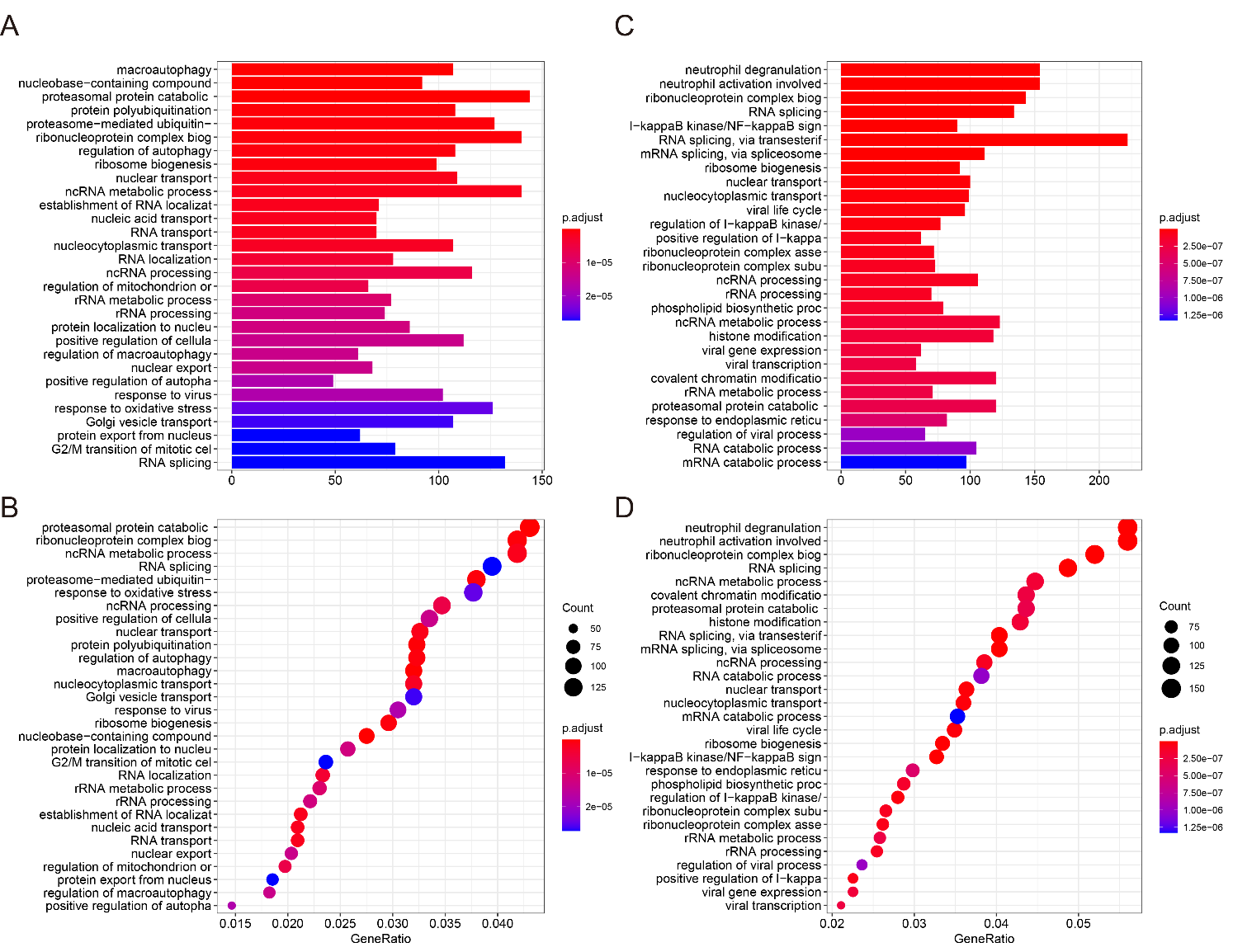


**Figure S16** GO enrichment analysis of macrophages’ differentially expressed genes in melanoma. **(A-B)** GO enrichment analysis of down-regulated genes in high risk score melanoma-infiltrated macrophages. **(C-D)** GO enrichment analysis of up-regulated genes in high risk score melanoma-infiltrated macrophages.


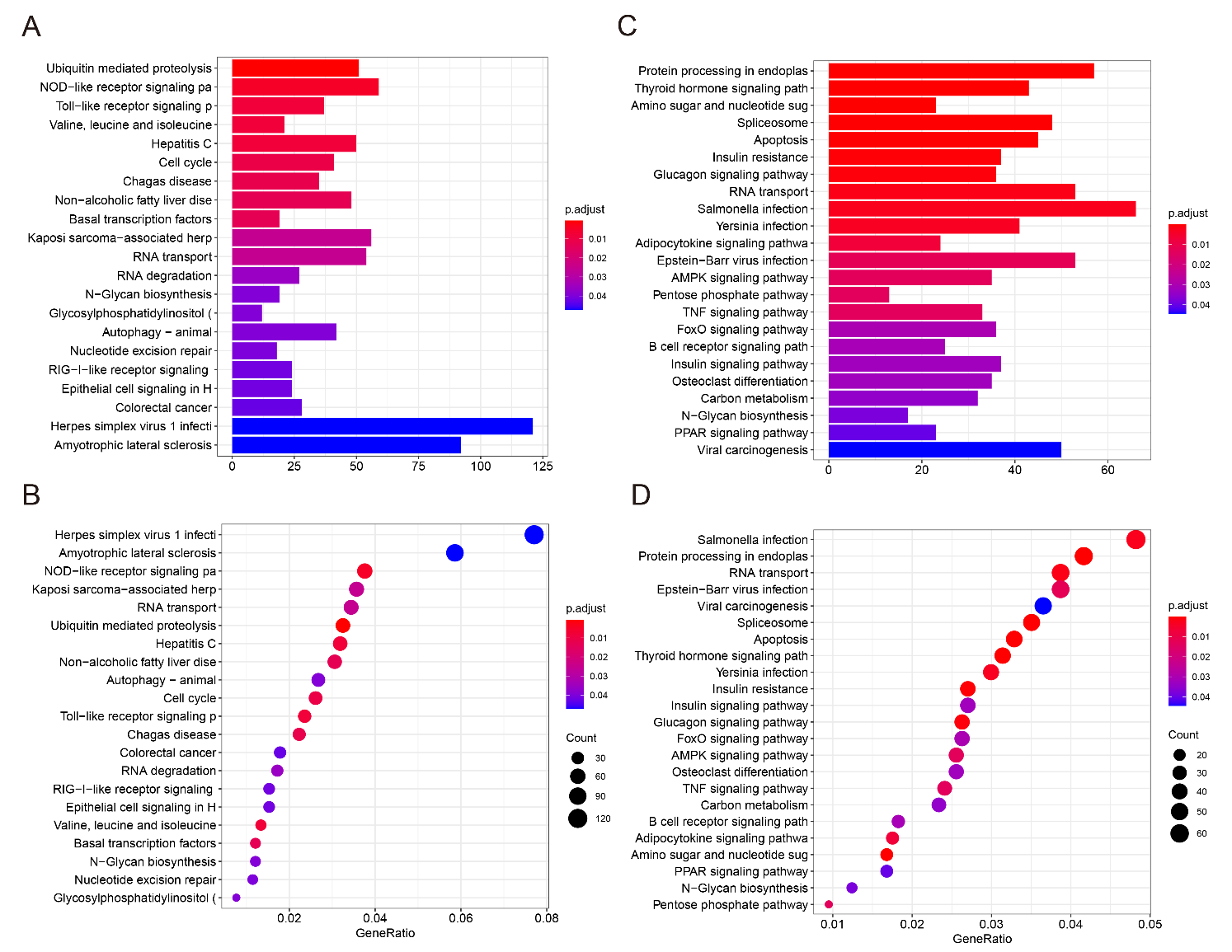


**Figure S17** KEGG enrichment analysis of macrophages’ differentially expressed genes in melanoma. **(A-B).** KEGG enrichment analysis of down-regulated genes in high risk score melanoma-infiltrated macrophages. c-d). KEGG enrichment analysis of up-regulated genes in high risk score melanoma-infiltrated macrophages.
