# Supplementary material for: Pattern Recognition Receptors (PRRs) in Macrophages Possess Prognosis and Immunotherapy Potential for Melanoma
Source: Front Immunol. 2021 Nov 9;12:765615. doi: 10.3389/fimmu.2021.765615 (PMC8630683; doi:10.3389/fimmu.2021.765615)
Supplement: Supplementary file 2 [file Table_1.docx]

Supplementary Tables

Pan-cancer and Single-cell Transcriptome Analyses Identify Pattern Recognition Receptors (PRRs) in Macrophages with Prognosis and Immunotherapy Potential for Melanoma

Table S1 SKCM prognosis-related PRR genes and their p(HR) values.

| Gene | Protein Name | Type | p(HR) in SKCM |
| --- | --- | --- | --- |
| TLR1 | Toll-like receptor 1 | TLR | 0.0021 |
| TLR2 | Toll-like receptor 2 | TLR | 1.20E-06 |
| TLR3 | Toll-like receptor 3 | TLR | 0.015 |
| TLR4 | Toll-like receptor 4 | TLR | 5.00E-06 |
| TLR5 | Toll-like receptor 5 | TLR | 0.056 |
| TLR6 | Toll-like receptor 6 | TLR | 0.072 |
| TLR7 | Toll-like receptor 7 | TLR | 4.00E-04 |
| TLR8 | Toll-like receptor 8 | TLR | 9.60E-06 |
| TLR9 | Toll-like receptor 9 | TLR | 0.39 |
| TLR10 | Toll-like receptor 10 | TLR | 6.30E-05 |
| MB21D1 | Cyclic GMP-AMP synthase | DNA Sensor | 0.058 |
| DDX60 | Probable ATP-dependent RNA helicase DDX60 | DNA Sensor | 1.80E-05 |
| ZBP1 | Z-DNA-binding protein 1 | DNA Sensor | 8.70E-06 |
| AIM2 | Interferon-inducible protein AIM2 | DNA Sensor | 0.00023 |
| DDX58 | Antiviral innate immune response receptor RIG-I | RNA Sensor | 0.00014 |
| IFIH1 | Interferon-induced helicase C domain-containing protein 1 | RNA Sensor | 0.00043 |
| NOD1 | Nucleotide-binding oligomerization domain-containing protein 1 | NLR | 0.24 |
| NOD2 | Nucleotide-binding oligomerization domain-containing protein 2 | NLR | 7.50E-05 |
| NLRP3 | NACHT, LRR and PYD domains-containing protein 3 | NLR | 0.0015 |
| NLRC4 | NLR family CARD domain-containing protein 4 | NLR | 1.60E-06 |

Table S2 Immune signaling genes and their p(HR) value in SKCM.

| Gene | Protein Name | p(HR) in SKCM |
| --- | --- | --- |
| IRF1 | Interferon regulatory factor 1 | 1.10E-06 |
| IRF7 | Interferon regulatory factor 7 | 0.00055 |
| CARD9 | Caspase recruitment domain-containing protein 9 | 0.003 |
| NFKB2 | Nuclear factor NF-kappa-B p100 subunit | 0.037 |
| NFKB1 | Nuclear factor NF-kappa-B p105 subunit | 0.078 |
| MAL | Myelin and lymphocyte protein | 0.13 |
| TRAF6 | TNF receptor-associated factor 6 | 0.14 |
| BCL10 | B-cell lymphoma/leukemia 10 | 0.15 |
| MAVS | Mitochondrial antiviral-signaling protein | 0.15 |
| MYD88 | Myeloid differentiation primary response protein MyD88 | 0.21 |
| TRAF3 | TNF receptor-associated factor 3 | 0.21 |
| TICAM1 | TIR domain-containing adapter molecule 1 | 0.21 |
| IRF3 | Interferon regulatory factor 3 | 0.27 |
| TAB2 | TGF-beta-activated kinase 1 and MAP3K7-binding protein 2 | 0.28 |
| TBK1 | Serine/threonine-protein kinase TBK1 | 0.46 |
| IKBKB | Inhibitor of nuclear factor kappa-B kinase subunit beta | 0.61 |
| TAB3 | TGF-beta-activated kinase 1 and MAP3K7-binding protein 3 | 0.71 |
| CHUK | Inhibitor of nuclear factor kappa-B kinase subunit alpha | 0.72 |
| TMEM173 | Stimulator of interferon genes protein | 0.91 |
| RIPK2 | Receptor-interacting serine/threonine-protein kinase 2 | 0.94 |

Table S3 PRR-induced inflammation-related genes and their p(HR) value in SKCM.

| Gene | Protein name | p(HR) in SKCM |
| --- | --- | --- |
| Casp5 | Caspase-5 | 3.10E-05 |
| Casp4 | Caspase-4 | 0.0089 |
| Casp1 | Caspase-1 | 0.84 |
| GSDMD | Gasdermin-D | 0.0097 |
| CASP10 | Caspase-10 | 0.0047 |
| CASP9 | Caspase-9 | 0.77 |
| CASP7 | Caspase-7 | 0.038 |
| CASP3 | Caspase-3 | 0.089 |
| GZMB | Granzyme B | 1.20E-05 |
| IL23A | Interleukin-23 subunit alpha | 0.38 |
| IL12A | Interleukin-12 subunit alpha | 6.20E-07 |
| IL12B | Interleukin-12 subunit beta | 0.024 |
| IL18 | Interleukin-18 | 1.90E-06 |
| IL1A | Interleukin-1 alpha | 0.83 |
| IL1B | Interleukin-1 beta | 2.10E-02 |
| IL6 | Interleukin-6 | 0.24 |
| IFNG | Interferon gamma | 1.30E-05 |
| IFNB1 | Interferon beta | N/A |
| IFNA2 | Interferon alpha-2 | N/A |
| IFNA1 | Interferon alpha-1/13 | N/A |
| TNF | Tumor necrosis factor | 0.0038 |

Table S4 Details of single-cell transcriptome data.

|  | SKCM | LIHC | BRCA | PAAD |
| --- | --- | --- | --- | --- |
| Data source | GEO | CNGB | GEO | GEO |
| serial number | GSE72056 | CSE0000008 | GSE176078 | GSE111672 |
| Platforms | Illumina NextSeq 500 (Homo sapiens) | 10x Genomics Single Cell 50 Library Construction Kit; 10x Genomics Single Cell V(D)J Enrichment Kit, Human T Cell;  Illumina TruSeq RNA Access Library Prep Kit | Illumina NextSeq 500 (Homo sapiens) | Illumina NextSeq 500 (Homo sapiens) |
| samples count | 19 | 19 | 26 | 23 |
